# Molecular Diagnosis of Leishmaniasis: Development of a qPCR Assay for Genus Detection and Differentiation of *Leishmania* (L.) *amazonensis* and *Leishmania* (V.) *braziliensis*

**DOI:** 10.3390/diagnostics16111704

**Published:** 2026-06-02

**Authors:** Guilherme Ferreira Correia, Bruna Terci Fernandes, Paulo Henrique Guilherme Borges, Isabela Madeira de Castro, Guilherme Bartolomeu-Gonçalves, Thiago França Soares, Eloiza Teles Caldart, Phileno Pinge-Filho, Ivete Conchon-Costa, Vitor Takashiba, Nayara Anitelli Artero, Marco Aurélio Fornazieri, Wander Rogério Pavanelli, Eliandro Reis Tavares, Lucy Megumi Yamauchi, Celso Vataru Nakamura, Sueli Fumie Yamada-Ogatta

**Affiliations:** 1 Programa de Pós-Graduação em Microbiologia, Universidade Estadual de Londrina, Londrina CEP 86057-970, Paraná, Brazil; guilhermeferreiracorreia@gmail.com (G.F.C.); paulo.guilhermeph@uel.br (P.H.G.B.); isabela.mcastro@uel.br (I.M.d.C.); thiago.franca.soares01@gmail.com (T.F.S.); pingefilho@uel.br (P.P.-F.); tavares.eliandro@uel.br (E.R.T.); lionilmy@uel.br (L.M.Y.); 2 Departamento de Farmácia, Faculdade Cristo Rei, Cornélio Procópio CEP 86300-000, Paraná, Brazil; terci.bruna@gmail.com; 3 Laboratório de Biologia Molecular de Microrganismos, Departamento de Microbiologia, Universidade Estadual de Londrina, Londrina CEP 86038-350, Paraná, Brazil; guilherme.bartolomeu@uel.br; 4 Departamento de Medicina Veterinária Preventiva, Universidade Estadual de Londrina, Londrina CEP 86057-970, Paraná, Brazil; eloizacaldart@uel.br; 5 Programa de Pós-Graduação em Patologia Experimental, Universidade Estadual de Londrina, Londrina CEP 86057-970, Paraná, Brazil; conchon@uel.br (I.C.-C.); wanderpavanelli@uel.br (W.R.P.); 6 Departamento de Clínica Cirúrgica, Universidade Estadual de Londrina, Londrina CEP 86038-350, Paraná, Brazil; vitortakashiba@gmail.com (V.T.); nayara.anitelli@uel.br (N.A.A.); marcofornazieri@gmail.com (M.A.F.); 7 Laboratório de Inovação Tecnológica no Desenvolvimento de Fármacos e Cosméticos, Universidade Estadual de Maringá, Maringá CEP 87020-900, Paraná, Brazil

**Keywords:** American tegumentary leishmaniasis, internal transcribed spacer 2, kDNA minicircles, melting curve analysis, real-time PCR, nucleic acid amplification test

## Abstract

**Background/Objective**: Leishmaniasis is a neglected tropical disease caused by species of the genus *Leishmania*, with a broad clinical spectrum that can overlap with other infectious and non-infectious conditions. Accurate species identification is critical for appropriate treatment and prognosis; however, parasitological methods are limited by suboptimal sensitivity, specificity, and inability to reliably differentiate species. This study aimed to develop and validate a real-time PCR assay based on melting-curve analysis (Leish-qPCR) for the detection of *Leishmania* spp. and the differentiation of *Leishmania* (*Leishmania*) *amazonensis* and *Leishmania* (*Viannia*) *braziliensis*. **Methods and Results**: Genus-specific primers were designed based on the kDNA (kinetoplast DNA) minicircle consensus sequences of *Leishmania* species, while species-specific primers targeted the internal transcribed spacer 2 (ITS2) consensus regions of the ribosomal RNA locus of *L.* (L.) *amazonensis* and *L.* (V.) *braziliensis*. Analytical performance was evaluated in silico and in vitro using a panel of protozoa, fungi, and bacteria, exhibiting 100% specificity with no cross-amplification. The limit of detection was one copy per reaction for all targets using positive controls. Clinical validation was performed using skin biopsy specimens from patients with granulomatous lesions. The optimized Leish-qPCR assay, performed in separate reaction tubes within the same run, demonstrated reliable analytical specificity and sensitivity, with distinct and reproducible melting temperature (Tm) peaks across plasmid controls, parasite DNA, and clinical samples. Comparative analysis with histopathological examination demonstrated moderate agreement between the methods, supporting the applicability of the assay for sensitive detection and species-level discrimination of *Leishmania* spp. in clinical samples. **Conclusions**: The Leish-qPCR assay presented high sensitivity, specificity, and diagnostic accuracy, representing a promising tool for routine diagnosis of leishmaniasis and for the differentiation of *L.* (L.) *amazonensis* and *L.* (V.) *braziliensis* in clinical samples.

## 1. Introduction

Leishmaniasis is a neglected tropical disease caused by protozoa of the genus *Leishmania* (Kinetoplastida order, *Trypanosomatidae* family) transmitted by infected blood-sucking female sand flies. The pathogenesis of leishmaniases is associated with a broad spectrum of clinical manifestations, ranging from asymptomatic infection to chronic, debilitating disease. Leishmaniases are traditionally grouped into three main forms: cutaneous leishmaniasis (CL), mucocutaneous leishmaniasis (MCL), and visceral leishmaniasis (VL) also known as kala-azar. CL is the most common form of the disease, while VL is considered the most severe form and is often fatal in the absence of adequate treatment; MCL stands out for its high disabling potential as well as functional and psychosocial impact on affected individuals [1,2].

Currently, it is estimated that more than 1 billion people live in areas endemic for leishmaniases and are at risk of infection. Annually, there are approximately 30,000 new cases of VL and more than 1 million new cases of CL worldwide, constituting a major global public health problem. In this context, Brazil is among the countries with the highest burden of leishmaniasis, characterized by a high incidence and broad geographic distribution of its clinical forms [1]. The country has reported approximately 307,440 cases over the past two decades, corresponding to an estimated mean incidence of 8.96 cases per 100,000 inhabitants, underscoring its continued epidemiological relevance and impact on public health [3].

Approximately 20 *Leishmania* species infect humans [4,5], with *Leishmania* (*Viannia*) *braziliensis* and *Leishmania* (*Leishmania*) *amazonensis* being the most prevalent in Brazil [3]. Notably, *L.* (V.) *braziliensis* has the widest geographic distribution and greatest clinical relevance, accounting for most cases of American tegumentary leishmaniasis (ATL) in the country. It is frequently associated with ulcerative cutaneous lesions and, importantly, mucocutaneous disease. Globally, this species is a major etiological agent of MCL in the Americas, highlighting the importance of accurate species-level diagnosis for appropriate clinical management [1,6,7].

*L*. (L.) *amazonensis* is predominantly distributed across the American continent, with the highest occurrence in the Amazon Basin, specifically in Brazil, Guyana, and Venezuela. This species is commonly associated with both localized cutaneous and diffuse CL, a rare but severe manifestation characterized by the spread of multiple nodular lesions and deficiency in the host’s cellular immune response [1,5,6].

The outcome of leishmaniasis depends on early diagnosis and accurate *Leishmania* species identification, as therapeutic strategies vary according to the etiological agent [2,6]. However, the disease presents a broad spectrum of clinical manifestations that are not species-specific and often overlap with other infectious and non-infectious conditions, thereby limiting the reliability of clinical diagnosis alone [8].

The diagnostic gold standard remains the direct detection of the parasite through conventional methods such as culture, lesion smears/imprints, and histopathology [1,3,9]. Culture enables parasite isolation but is time-consuming, while histopathology relies on identifying amastigotes in tissue sections, typically within macrophages in a granulomatous inflammatory background [10]. Despite routine use, these methods show limited sensitivity, especially in samples with low parasite burdens, and do not reliably allow for species-level differentiation, which is essential for appropriate management [9]. Therefore, there is a need for sensitive and specific methods capable of both detecting *Leishmania* spp. and discriminating clinically relevant species. In line with the World Health Organization Roadmap for Neglected Tropical Diseases 2021–2030, which highlights diagnosis as a cornerstone for disease control [11], this study addresses these limitations by developing a real-time PCR assay for both detection and species differentiation.

PCR-based molecular diagnostics provide higher sensitivity and specificity, enabling the detection of parasite DNA even in samples with low parasitic loads [9,12,13,14,15,16]. Importantly, these methods also allow for accurate species identification, supporting both clinical management and epidemiological surveillance. Several genomic regions of *Leishmania* spp. have been investigated as targets for the diagnosis of leishmaniases. Among the molecular targets, kinetoplast DNA (kDNA) minicircles are widely used due to their high copy number (approximately 5000–10,000 per cell) [17], which enhances analytical sensitivity [15,18,19,20,21,22,23,24,25,26,27]. In addition, their conserved and variable regions enable both broad detection and species-specific approaches [28,29].

For species discrimination, the internal transcribed spacer (ITS) region of the ribosomal RNA (rRNA) locus has emerged as a robust target [15,30,31,32]. ITS sequences exhibit extensive sequence variability, are relatively short (approximately 1 kb in *Leishmania* spp.) and are flanked by highly conserved segments [33,34]. Additionally, in *Leishmania* spp., an estimated 20–200 copies of the rRNA unit are tandemly repeated per haploid genome [35,36,37]. ITS sequences combine high interspecific variability with conserved flanking regions, facilitating reliable PCR amplification and differentiation among *Leishmania* species. Together, these molecular targets support the development of sensitive and specific assays tailored for simultaneous detection and species-level identification [33,34,36,37].

This study aimed to develop a real-time PCR assay based on melting curve analysis (Leish-qPCR) for the simultaneous detection of *Leishmania* spp. and the differentiation of *L.* (L.) *amazonensis* and *L.* (V.) *braziliensis*. The assay targets minicircle kDNA regions for genus-level detection and the ITS2 region for species-specific identification. Its performance was evaluated using skin biopsy samples. Although ITS2 has been previously explored for *Leishmania* identification, this study is, to our knowledge, the first to apply SYBR Green-based qPCR coupled with melting curve analysis for the discrimination of these two species.

## 2. Materials and Methods

### 2.1. Oligonucleotide Primers and Positive Controls

The nucleotide sequences of the conserved region of the minicircles of *Leishmania* spp. kDNA and of the ITS2 region of the rRNA locus of *L.* (V.) *braziliensis* and *L.* (L.) *amazonensis* were retrieved from the GenBank/EMBL databases (available at http://www.ncbi.nlm.nih.gov, accessed on 10 July 2024). For the genus *Leishmania* and for each species, all sequences were subjected to multiple sequence alignments using the ClustalW algorithm of the BioEdit v.7.2.0 software (Supplementary Table S1) to obtain a consensus sequence representative of the genus *Leishmania* and each species.

Genus- and species-specific primers were designed based on the consensus sequences of each target region using the PrimerQuest™ and OligoAnalyzer™ tools (both available at http://www.idtdna.com, accessed on 12 July 2024). To enhance assay specificity and accuracy, the selection of target nucleotide sequences was guided by the following criteria: (i) selection of essential genes; (ii) conservation of sequences within each species to minimize false-negative results arising from genetic variability; and (iii) absence of significant secondary structures or primer-dimer formation (self- and heterodimers), with predicted values not exceeding ΔG values above −9 kcal/mol and melting temperature (Tm) variations ≤ 10%.

To ensure specificity, the primer sequences were compared with the nucleotide sequences available in the GenBank database of the National Center for Biotechnology Information (accessed on 12 July 2024) using the BLAST algorithm (blastn). Additionally, primers targeting the ribonuclease P (RNase P) gene involved in human transfer RNA (tRNA) processing [38] were included in this study. The characteristics of the primer sequences and expected amplicon sizes are presented in Table 1. To obtain positive controls, the consensus sequences of each target (Supplementary Table S2) were inserted into the pUC57 plasmid (FastBio Ltda., Ribeirão Preto, Brazil).

### 2.2. Protozoal and Microbial Strains

A panel composed of eight protozoan species, as well as 14 fungal species, nine bacterial species, and human DNA (Table 2), was used to develop the assays. This panel included different microbial species representative of the nasopharyngeal microbiota, as well as potential pathogens associated with granulomatous infections.

The promastigote forms of *Leishmania* species were cultured in Warren medium (brain-heart infusion supplemented with 10 µg/mL hemin and 10 µg/mL folic acid; pH 7.2), supplemented with 10% heat-inactivated fetal bovine serum (FBS), and incubated at 25 °C for 72 h. Epimastigote forms of *Trypanosoma cruzi* Y strain [39] were cultured in liver-tryptose infusion medium, pH 7.4 [40], supplemented with 10% heat-inactivated FBS at 28 °C for 72 h.

Three colonies of each bacterial and fungal species were cultured at 37 °C for 24 h in trypticase soy broth (Oxoid, São Paulo, Brazil) and Sabouraud dextrose broth (Himedia, Thane, India), respectively.

After cultivation, the microbial cells were collected by centrifugation (10,000× *g* for 5 min), washed twice with sterile 0.15 M phosphate-buffered saline, pH 7.2, and processed for DNA purification. The microorganisms were stored at −80 °C in their respective culture media containing 20% glycerol.

### 2.3. DNA Purification

DNA purification was performed using the QIAamp^®^ DNA Mini Kit (QIAGEN, São Paulo, Brazil), following the manufacturer’s instructions. DNA concentration was determined using a BioTek Synergy™ HT microtiter plate reader spectrophotometer at 260 nm (Agilent, Santa Clara, CA, USA). After quantification, the DNA from each microorganism was adjusted to a final concentration of 50 ng/µL.

### 2.4. Real-Time PCR Design

The amplification reaction conditions for *Leishmania* molecular targets were defined in two stages. Initially, the annealing temperatures and primer concentrations were determined using conventional PCR. Then, the optimized conditions were evaluated in qPCR assays.

For conventional PCR, each pair of primers, at concentrations ranging from 0.5 to 2 μM, was used in amplification reactions with a final volume of 20 μL. The reaction mixture contained 20 mM Tris-HCl (pH 8.4), 50 mM KCl, 1.5 mM MgCl_2_, 100 μM of each dNTP, 1 U of Invitrogen™ *Taq* DNA polymerase (ThermoFisher Scientific, São Paulo, Brazil), and 1 × 10^6^ copies of the positive controls. Amplifications were performed in a Veriti 96-well thermocycler (Applied Biosystems, São Paulo, Brazil), with an initial denaturation step at 95 °C for 1 min, followed by 35 cycles of denaturation at 95 °C for 30 s, annealing at a temperature gradient ranging from 60 °C to 70 °C for 1 min, and extension at 72 °C for 45 s. Negative template control (NTC) reactions, without the addition of nucleic acids, were included in all runs. The amplified products were analyzed by electrophoresis on a 3% agarose gel after staining with GelRed^®^ (Biotium-Uniscience, Osasco, Brazil).

Based on these results, an annealing temperature of 62 °C and a primer concentration of 1 μM were selected for validation of the qPCR assays, using both positive controls and purified nucleic acids from *L.* (L.) *amazonensis* and *L.* (V.) *braziliensis* as templates. The *Leishmania* real-time polymerase chain reaction assays (hereafter, this assay is referred to as the Leish-qPCR assay) were performed on a Rotor-Gene Q 5Plex HRM (QIAGEN, Hilden, Germany), in a final volume of 20 µL, which contained 1 × 10^6^ copies of the positive controls, 1 μM of each pair of primers complementary to the protozoan targets, 1 μM of primer pair targeting the human RNase P encoding gene, and the QuantiNova SYBR^®^ Green PCR mixture (QIAGEN, São Paulo, Brazil), according to the manufacturer’s recommendations.

The cycling conditions consisted of an initial denaturation at 95 °C for 2 min, followed by 40 cycles of 95 °C for 30 s, 62 °C for 30 s, and 72 °C for 30 s. Melting curves were generated using temperature increments of 0.5 °C, with a 60-s hold at each step, over a temperature range of 65 °C to 99 °C. NTC reactions were run in parallel. Data were analyzed using the Rotor-Gene Q Series software, version 2.1.0.9.

### 2.5. Analytical Specificity and Sensitivity

The specificity of the Leish-qPCR assay was evaluated using 100 ng of nucleic acids extracted from cultures of a panel of protozoa, bacteria, and fungi (Table 2). All amplification reactions were performed in duplicate across three independent experimental runs.

The analytical sensitivity of the Leish-qPCR assay was empirically determined using serial dilutions of quantified positive controls [41,42], along with purified DNA from *L.* (L.) *amazonensis* and *L.* (V.) *braziliensis* as templates. The assay covered a dynamic range from 10^0^ to 10^6^ copies and from 10^0^ to 10^5^ parasite equivalents per reaction. Each dilution was tested in six replicates over five consecutive days to assess intra-assay and inter-assay variability. The limit of detection (LoD) was defined as the lowest target concentration consistently detected in ≥95% of replicates, with reproducible Ct values and acceptable variance. For each primer pair, a standard curve was generated by plotting the mean Ct values against the input copy or parasite equivalent number. The coefficient of determination (R^2^) was calculated to assess assay linearity, and the amplification efficiency (E) was derived from the slope of the standard curve using the equation: E = 10^−1/slope^ − 1.

### 2.6. Performance of Real-Time PCR in Clinical Samples

The clinical specimens (*n* = 60) analyzed in this study consisted of skin biopsy fragments obtained from the nasopharyngeal region, upper and lower limbs, hands, and ears of patients presenting with granulomatous lesions and treated at the Specialty Outpatient Clinic of the University Hospital of Universidade Estadual de Londrina (UEL), Londrina, Paraná State, Brazil. Sample collection was conducted in strict accordance with Good Clinical Practice guidelines by trained and qualified physicians at the University Hospital of UEL, ensuring high standards of quality, patient safety, and specimen integrity.

Samples were collected between July 2022 and May 2026 in compliance with the ethical standards established by the Ethics Committee of the UEL (Document 57452322.2.0000.5231 and opinion number 5.406.144). All participants provided written informed consent by signing the Informed Consent Form, confirming their voluntary participation and ensuring the protection of their rights.

Genomic DNA was extracted from skin biopsy specimens using the QIAamp DNA Mini Kit (QIAGEN, São Paulo, Brazil), following the manufacturer’s protocol. The samples were digested with proteinase K in lysis buffer at 56 °C for 1–3 h until complete tissue solubilization. The resulting lysate was then processed according to the kit instructions. After quantification, as above, the DNA from each sample was adjusted to a final concentration of 20 ng/µL, and 5 µL was used in Leish-qPCR assays. The samples were also subjected to a qPCR assay targeting the kDNA minicircle region, using previously described and validated primers JW11 (5’ CCTATTTTACACCAACCCCCAGT 3’) and JW12 (5’ GGGTAGGGGCGTTC TGCGAAA 3’) [18] as a complementary molecular approach to support the analysis.

### 2.7. Diagnostic Agreement Between Real-Time PCR and Histopathology Methods Using the Kappa Coefficient

To assess the agreement between Leish-qPCR and histopathological techniques, Cohen’s kappa coefficient (κ) and its corresponding 95% confidence interval (95% CI) were calculated. Data from 60 patients were analyzed considering binary categorical variables corresponding to the results obtained by each diagnostic method. Statistical analyses were performed using the Python programming language, version 3.13, specifically the sklearn.metrics library. Confidence intervals were estimated using standard binomial methods. Concordance matrices were generated to visualize the performance of the assay relative to the reference method.

The interpretation of kappa values followed the criteria proposed by Landis and Koch [43]: values ≤ 0.20 indicate slight agreement; 0.21–0.40 indicate fair agreement; 0.41–0.60 indicate moderate agreement; 0.61–0.80 indicate substantial agreement; and ≥0.81 indicate almost perfect agreement.

Additionally, McNemar’s test was applied to evaluate differences between the paired results obtained by the two diagnostic techniques, with *p*-values < 0.05 considered statistically significant.

## 3. Results

### 3.1. Melting-Curve-Based Real-Time PCR Assay

The nucleotide sequences of the kDNA minicircle and the ITS2 region of the rRNA locus from *Leishmania* spp., as well as from *L.* (L.) *amazonensis* and *L.* (V.) *braziliensis*, respectively, were retrieved from the GenBank nucleotide database and analyzed to identify consensus regions (Supplementary Table S1). These consensus sequences were used for the design of genus-specific (kDNA) and species-specific (ITS2) primers (Table 1), and to construct a synthetic plasmid that was used as a positive control (Supplementary Table S2). The similarity of each primer pair to other nucleotide sequences was assessed using the BLAST algorithm (v. 2.15.0). No significant sequence similarity was identified other than those corresponding to the intended *Leishmania* genus and species targets. Furthermore, no matches were detected against the human genome. These findings suggest a low likelihood of cross-reactivity with non-target sequences.

**Figure 1:**
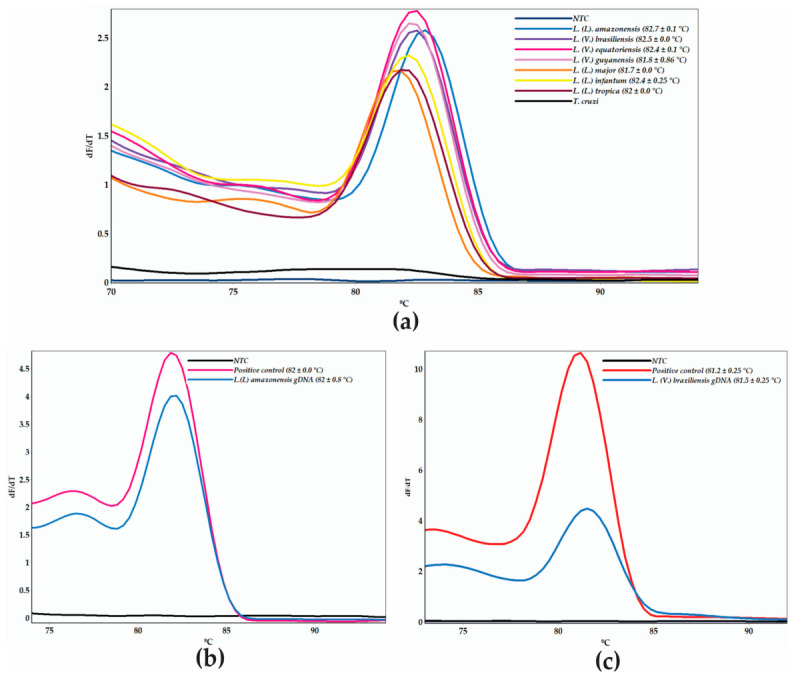
Representative melting curve analyses showing the melting temperature (Tm) peaks of the (**a**) kDNA minicircle region for the *Leishmania* genus, and (**b**) the ITS2 region of *Leishmania* (L.) *amazonensis* and (**c**) *Leishmania* (V.) *braziliensis*, using genomic DNA (gDNA) from the parasites and the plasmid positive control.

The target amplification conditions were standardized in two steps. Initially, annealing temperature and primer concentrations were optimized using conventional monoplex PCR. Using synthetic plasmids as positive controls, all primer pairs (1 µM) generated amplicons of the expected sizes (Table 1) at an annealing temperature of 62 °C. These conditions were further assessed in monoplex qPCR assays to determine the melting temperature (Tm) of each primer pair. All targets were successfully amplified, yielding dissociation curves with a single peak, indicative of specific amplification. The Tm values obtained for all amplicons are presented in Table 1 and Figure 1. The Tm values were further confirmed in monoplex qPCR assays using purified DNA from cultures of *Leishmania* species (Figure 1). Similar Tm values were observed for the ITS2 regions of *L.* (L.) *amazonensis* and *L.* (V.) *braziliensis* compared with those obtained using the plasmid controls. For the kDNA target, Tm values ranged from 81.7 ± 0.00 °C for *L.* (L.) *major* to 82.7 ± 0.10 °C for *L.* (L.) *amazonensis*, which was also consistent with the value obtained using the plasmid positive control (Figure 1).

In addition, to assess nucleic acid purification quality and the presence of potential PCR inhibitors, primers targeting the human RNAse P-encoding gene [38] were included. Amplification of this target yielded a mean Tm value of 83.1 ± 0.20 °C (Table 1, Supplementary Figure S1).

### 3.2. Analytical Performance of the Assay

The specificity of the Leish-qPCR assay was experimentally validated using nucleic acids from a panel of protozoa, bacteria and fungi (Table 2). Amplification signals were detected exclusively for *Leishmania* species, indicating the absence of cross-reactivity with non-target organisms.

**Figure 2:**
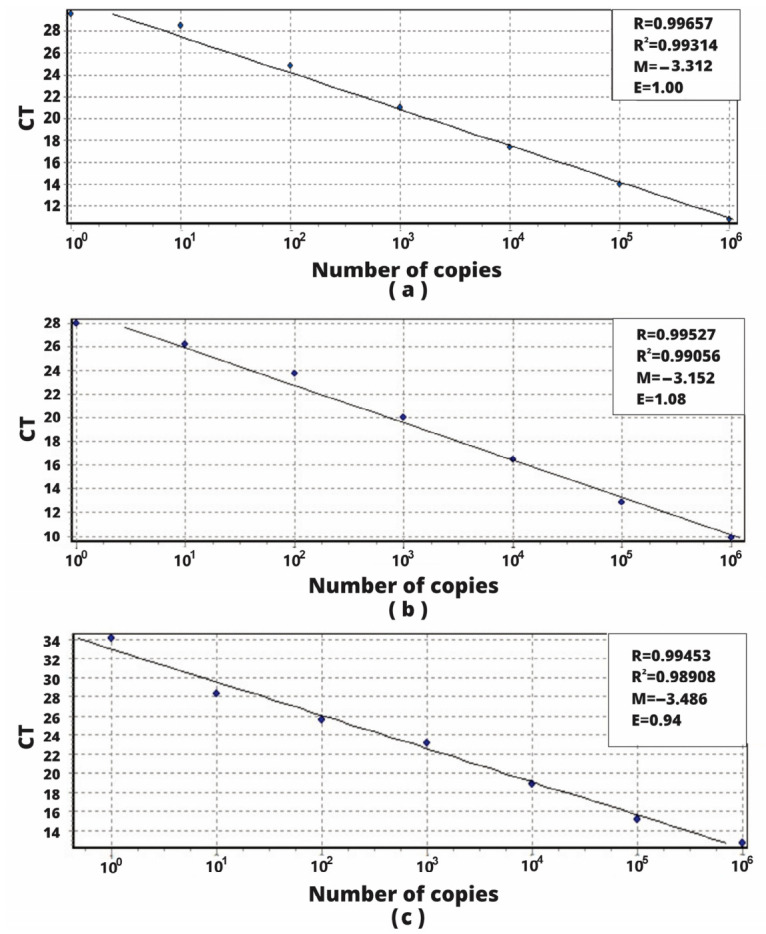
Sensitivity of Leish-qPCR assays. (**a**) kDNA minicircle of *Leishmania* spp.; (**b**) ITS2 of *L.* (L.) *amazonensis*; (**c**) ITS2 of *L.* (V.) *braziliensis*. Amplification plots of 10-fold serial dilutions corresponding to 10^0^–10^6^ copies of the positive controls. Standard curves were generated by linear regression analysis of threshold cycle (Ct) versus copy numbers. The slope (M), regression coefficient (R), and amplification efficiency of the qPCR assay are shown (**a**–**c**). The analyses were performed using the Rotor-Gene Q Series instrument and processed with the Rotor-Gene Q Series Software version 2.1.0.9. based on the Ct values obtained during amplification.

The analytical linearity, limit of detection (LoD), repeatability, and reproducibility of the Leish-qPCR assay targeting the selected genomic regions were evaluated using tenfold serial dilutions of positive controls, ranging from 10^6^ to 10^0^ copies per reaction. Each dilution was tested in six replicates per run over five independent days (*n* = 30), allowing for the assessment of intra-assay (repeatability) and inter-assay (reproducibility) precision [42]. The LoD for all molecular targets was established at one copy per reaction (Figure 2), reflecting the high analytical sensitivity of the assay. This performance is consistent with previously reported LoD values for multicopy molecular targets within the *Leishmania* genome [22,23,25,26,31,32].

Amplification efficiencies, calculated from the slopes of the standard curves, ranged from 94% to 108%, which is within the acceptable range recommended for qPCR assays [42]. The kDNA-based assay demonstrated high linearity (R^2^ = 0.99314; R = 0.99657), with a slope of −3.312 and an amplification efficiency of 100% (Figure 2a). The ITS2-based assays also showed adequate linearity and amplification performance. For *L.* (L.) *amazonensis*, the assay exhibited R^2^ = 0.99056 and R = 0.99527, with a slope of −3.152 and an amplification efficiency of 108% (Figure 2b). For *L.* (V.) *braziliensis*, the standard curve showed R^2^ = 0.98908 and R = 0.99453, with a slope of −3.486 and an amplification efficiency of 94% (Figure 2c). These findings are consistent with the high copy number of kDNA minicircles (approximately 5000–10,000 copies per cell) [17] and the moderate copy number of rRNA gene units (estimated at 20–200 copies per genome) [35,36,37] present in the parasite, both of which contribute to enhanced diagnostic sensitivity.

Mean Ct values and intra-assay standard deviations (SDs) of the dilution series of positive controls are shown in Table 3. Precision analysis demonstrated low variability across inter-assay runs, with coefficients of variation (CV%) for Ct values remaining below 2.9% at all concentrations tested. The CV% values at the LoD were 2.3%, 1.4% and 1.4% for *Leishmania* spp. kDNA, *L.* (L.) *amazonensis* ITS2 and *L.* (V.) *braziliensis* ITS2, respectively.

Purified DNA from cultures of *L.* (L.) *amazonensis* and *L.* (V.) *braziliensis* was used to evaluate the analytical linearity and LoD of the kDNA target in the Leish-qPCR assay under conditions more representative of routine clinical practice. DNA samples were subjected to 10-fold serial dilutions ranging from 10^5^ to 10^0^ parasite equivalents per reaction. Amplification efficiencies, calculated from the slopes of the standard curves, were 100% for *L.* (L.) *amazonensis* and *L.* (V.) *braziliensis*. An LoD of one parasite equivalent per reaction was achieved for both species (Supplementary Figure S2).

Based on these performance parameters, the assay was defined as positive for *Leishmania* genus and species when amplification of the corresponding molecular targets and the internal control was detected with Ct values ≤ 35 using the Rotor-Gene Q 5-Plex HRM System.

### 3.3. Performance of the Leish-qPCR Assay in Human Clinical Samples

The diagnostic performance of the Leish-qPCR assay was evaluated using skin biopsy specimens from 60 patients presenting with granulomatous lesions and treated at the University Hospital of UEL. Under the optimized reaction conditions, the assay generated reproducible amplification profiles for all targets, with a short overall turnaround time. Melting curve analysis demonstrated highly consistent Tm values across clinical samples for all targets. The mean Tm values (±SD) were 81.9 ± 0.14 °C for kDNA, 82.2 ± 0.01 °C for ITS2 of *L.* (L.) *amazonensis*, 81.2 ± 0.44 °C for ITS2 of *L.* (V.) *braziliensis*, and 82.8 ± 0.41 °C for RNase P. The observed Tm values closely matched those obtained from plasmid controls and reference parasite DNA, with only minor variations within a narrow range. No significant shifts in Tm were observed in clinical specimens, indicating that the assay is robust to potential inhibitors and variability associated with biological matrices. The RNase P internal control also exhibited stable Tm values, confirming the quality of DNA extraction and the absence of significant PCR inhibition.

Of the 60 clinical samples, 29 (48.3%) tested positive using kDNA-targeting primers, confirming the presence of *Leishmania* spp. The samples were also analyzed using an additional qPCR assay targeting the kDNA minicircle region, employing previously described and validated primers [18], as a supplementary molecular approach. All samples that tested positive with the Leish-qPCR assay were also positive with this assay.

Among the samples that tested positive for kDNA (*n* = 29), four (13.8%) were identified as *L.* (L.) *amazonensis* and 22 (75.9%) as *L.* (V.) *braziliensis*. Three kDNA-positive samples (10.3%) were not amplified by any species-specific primers, suggesting infection with other *Leishmania* species (Supplementary Table S3).

At the University Hospital of UEL, the routine diagnosis of leishmaniasis is based on histopathological examination of skin biopsy specimens for the detection of the parasite in tissue sections. Therefore, this method was adopted as the reference standard for comparison and validation of the results obtained with the Leish-qPCR assay developed in this study to reflect real-world clinical practice. Among the 60 samples analyzed, 14 were positive based on histopathological examination. The Leish-qPCR assay correctly identified all histopathology-positive samples, yielding a sensitivity of 100% (95% CI: 76.8–100%). Specificity was 67.4% (95% CI: 52.0–80.5%), with 15 samples testing positive by qPCR but negative by histopathology (Table 4).

The positive and negative predictive values were 48.3% (95% CI: 29.4–67.5%) and 100% (95% CI: 88.8–100%), respectively, with an overall accuracy of 75.0% (95% CI: 62.1–85.3%). Agreement between methods was moderate (Cohen’s κ = 0.49; 95% CI: 0.30–0.68, Supplementary Figure S3) according to the criteria proposed by Landis and Koch [43]. McNemar’s test revealed a statistically significant difference between the paired results obtained by the two methods (*p* < 0.001), which was driven by the higher number of qPCR-positive/histopathology-negative samples. This finding suggests that the molecular assay may have higher analytical sensitivity, potentially detecting infections with low parasite burdens not identifiable by conventional histopathological analysis.

Thus, the final optimized reaction conditions for the Leish-qPCR assay, which can be performed in separate tubes within the same run, consisted of a total reaction volume of 20 µL, which includes 1× QuantiNova™ SYBR^®^ Green mixture (QIAGEN, São Paulo, Brazil), 1.0 µM of each primer pair (including those of RNAse P), and 100 ng of purified DNA from clinical samples per reaction. Under these conditions, the amplification protocol included an initial denaturation step at 95 °C for 10 min, followed by 40 cycles of denaturation at 95 °C for 30 s, annealing at 62 °C for 30 s, and extension at 72 °C for 30 s, in a Rotor-Gene Q 5Plex HRM (QIAGEN, Hilden, Germany).

## 4. Discussion

In this study, a SYBR Green-based qPCR assay (Leish-qPCR) was developed to enable the simultaneous detection of *Leishmania* at the genus level and the discrimination of *L.* (L.) *amazonensis* and *L.* (V.) *braziliensis*, the main etiological agents of CL and MCL in Brazil and across Latin American countries [1,3]. The assay targets the minicircle kDNA region for genus-level detection and the ITS2 region of the rRNA locus for species-specific identification, while the RNase P gene serves as an internal control.

Importantly, melting curve analysis demonstrated highly consistent and reproducible Tm values for all targets across plasmid controls, reference parasite DNA, and clinical samples. The minimal variation observed across different matrices supports the robustness of the assay and confirms that melting curve-based discrimination remains reliable even in the presence of potential inhibitors and biological variability. This consistency is particularly relevant for SYBR Green-based approaches, in which specificity depends on the accurate resolution of distinct melting peaks. Taken together, these findings highlight the advantage of combining kDNA and ITS2 targets within a SYBR Green assay, enabling both sensitive detection and accurate species-level identification, which are critical for improving the diagnosis and clinical management of leishmaniasis.

In recent years, real-time PCR assays have emerged as promising molecular diagnostic tools for the direct detection of pathogens in clinical samples [13,14,15,19,20,21,23,24,30]. These approaches offer several advantages, including rapid turnaround time, high sensitivity, and the ability to simultaneously detect and differentiate multiple pathogens [13,14,15,16]. These features can facilitate early, targeted therapy and improve clinical management in patients with leishmaniasis.

The inclusion of the RNase P gene as an internal control represents a robust strategy for validating the quality of clinical samples and the efficiency of the DNA extraction process. As a ribonuclease involved in tRNA maturation and present in all nucleated human cells, amplification of RNase P gene confirms the presence of host genomic DNA and serves as an indicator of sample integrity [44]. The absence of RNase P amplification may indicate technical failure or inadequate sample collection, supporting result invalidation and reducing the risk of false-negative interpretations. This approach is particularly relevant for samples with low parasitic burdens, such as mucosal biopsies, cutaneous scrapings, and swabs [45].

Although other housekeeping genes, such those encoding β-actin [31] and GAPDH (glyceraldehyde-3-phosphate dehydrogenase) [19], have also been used as internal controls in diagnostic protocols for leishmaniasis, RNase P offers advantages related to its extensive validation in molecular assays, including protocols recommended by international reference organizations [38], as well as its consistent performance across different types of clinical samples [12,13].

Most qPCR assays developed for the detection of *Leishmania* spp. rely on hydrolysis probe-based detection systems [14,15,25,27], which require specialized instrumentation capable of discriminating multiple fluorophores, thereby increasing assay complexity and cost. In contrast, the use of the intercalating dye SYBR Green represents a robust, cost-effective, and widely accessible alternative, enabling target discrimination through melting curve analysis without the need for fluorescent probes [18,19,20,21,22,23,24,30,32].

In line with the present study, several investigations have used kDNA regions as molecular targets in combination with SYBR Green chemistry for the detection of *Leishmania* spp. For instance, Nicolas et al. [18] employed two primer sets (including the JW11 and JW12) targeting kDNA minicircle regions to detect and differentiate *L.* (L.) *major* (Tm = 83.18 °C), *L.* (L.) *donovani* (Tm = 83.26 °C), *L.* (L.) *tropica* (Tm = 83.03 °C), and *L.* (L.) *infantum* (Tm = 83.26 °C). However, the minimal differences in Tm values among these species, consistent with observations in the present study, limit reliable discrimination based solely on melting curve analysis. Similarly, De Paiva Cavalcanti et al. [21] employed kDNA-based amplification combined with melting curve analysis to identify *L.* (V.) *braziliensis* (Tm = 79.3 °C) in canine blood and human skin samples. However, as also observed in the present study, very similar Tm values were detected among different *Leishmania* species, corroborating the limited discriminatory power of melting curve analysis when used as the sole criterion for species identification.

Weirather et al. [19] evaluated 41 primer sets targeting multiple genomic regions of *Leishmania* spp., including kDNA minicircle sequences. Most primer combinations were assessed using both SYBR Green with melting curve analysis and hydrolysis probes (TaqMan). The SYBR Green-based assays demonstrated higher sensitivity for detecting the analyzed *Leishmania* species compared with the probe-based approach. Although species discrimination was achieved in some cases, overlapping Tm values among several species were again observed, consistent with the findings of Nicolas et al. [18], highlighting the intrinsic limitation of melting curve-based differentiation when target sequences exhibit high similarity.

The study by Gomes et al. [24] also employed kDNA minicircles in SYBR Green- and probe-based qPCR assays. Both approaches could discriminate between the *Leishmania* and *Viannia* subgenera, but the SYBR Green-based method showed higher accuracy. Consistently, other studies have also demonstrated the effectiveness of SYBR Green-based qPCR targeting kDNA for subgenus discrimination using melting curve analysis [20,22].

Other studies have indicated that approaches based on high-resolution melting (HRM) analysis can expand the discriminatory capacity of the kDNA-based qPCR assays. For example, Fayaz et al. [26] demonstrated that an HRM-based qPCR assay targeting kDNA minicircles can reliably differentiate *L.* (L.) *major*, *L.* (L.) *infantum*, and *L.* (L.) *tropica*. Similarly, Ceccarelli et al. [23] showed that a kDNA minicircle-targeting qPCR assay, combined with HRM analysis and minicircle abundance assessment, enables discrimination between *L.* (L.) *amazonensis* and *L.* (L.) *infantum*.

In this context, the ITS2 region was included in the present study as a complementary target to overcome the limitations of kDNA-based discrimination and improve species-level resolution. Although previous studies did not employ SYBR Green-based qPCR methodologies, they have highlighted the potential of ITS2 as an effective molecular marker for species-level discrimination of *Leishmania*. In particular, De Almeida et al. [30] proposed a PCR-based method followed by sequencing of the ITS2 region for species identification. This method was subsequently incorporated into the Centers for Disease Control and Prevention algorithm for the laboratory diagnosis of leishmaniasis. Nateghi Rostami et al. [31] developed a PCR assay targeting the ITS2 region to identify *L.* (L.) *major*, *L.* (L.) *tropica* and *L.* (L.) *infantum*. Species discrimination was based on differences in amplicon size visualized by agarose gel electrophoresis. In another study, Paun and Grigg [32] designed pan-genus primers anchored in the 5.8S and 28S ribosomal genes to amplify the highly polymorphic and size-variable ITS2 locus using a nested-PCR format, with species identification performed by sequencing the resulting amplicons generated by the internal primers of the assay.

While the Leish-qPCR assay standardized in this study demonstrated consistent analytical performance, some limitations should be acknowledged. (i) The assay was designed to only differentiate two species, *L.* (L.) *amazonensis* and *L.* (V.) *braziliensis*, which together account for the majority of leishmaniasis cases in Brazil and across Latin America [1,3]. Nevertheless, clinicians should be aware that other *Leishmania* species have been reported in clinical samples from these regions [46]. (ii) In the SYBR Green-based qPCR assay, species-specific amplicons exhibited highly similar melting temperatures, which may hinder unequivocal discrimination when targets are amplified simultaneously. To mitigate this limitation, reactions should be performed individually within the same run, preserving the simplicity and rapid turnaround of the assay. (iii) The LoD of the assay for all targets was determined using a synthetic plasmid positive control, which may not fully reflect sensitivity in clinical samples, as DNA extraction procedures can influence the assay performance. In addition, several matrix-related factors may influence assay performance in clinical specimens, including the presence of PCR inhibitors, DNA degradation, low and heterogeneous parasite burdens, and interference from host DNA and other biological components. Nevertheless, plasmid controls provide a standardized and reproducible approach for estimating analytical sensitivity in terms of target copy numbers, especially for multicopy targets such as rRNA repeat units and kDNA minicircles. To partially address this limitation and better reflect biologically relevant conditions, we additionally evaluated the assay sensitivity using purified parasite DNA, which incorporates the effects of genomic DNA complexity. Comparable amplification efficiencies were observed for the kDNA target, which was used as a representative marker, in both species. Future studies including spiked biological matrices and larger sets of clinical samples will be important to further validate assay performance under routine diagnostic conditions. (iv) The number of biological samples (*n* = 60) is relatively limited, which may affect the statistical robustness and generalizability of the findings. Therefore, further validation using larger and more diverse clinical cohorts is warranted to confirm the diagnostic performance of the assay. (v) The results of this study were compared exclusively with histopathological findings for the detection of *Leishmania*, which, although representing the routine diagnostic standard at the study site, may have inherent limitations in sensitivity.

Despite these limitations, the study highlights several advantages of the standardized Leish-qPCR assay. First, the complete workflow, including DNA extraction, sample preparation, and Leish-qPCR analysis, can be performed in 3 to 5 h, providing a rapid turnaround time, which is critical for timely diagnosis and patient treatment. Second, the estimated cost per skin biopsy sample, including reagents but excluding equipment and personnel, was approximately USD 6.50. Third, the assay can be easily adapted to conventional PCR platforms, making it suitable for laboratories without real-time PCR equipment. Finally, since the methodology relies on melting curve analysis, additional molecular targets can be incorporated into the assay, enabling future expansion to detect and differentiate a broader range of *Leishmania* species.

## 5. Conclusions

The SYBR Green-based real-time PCR assay combined with melting curve analysis developed in this study represents a cost-effective and technically accessible molecular diagnostic approach, with an optimized workflow that enables rapid results within 3 to 5 h. The assay allows for simultaneous genus-level detection (kDNA minicircles) and discrimination of *L.* (L.) *amazonensis* and *L.* (V.) *braziliensis* (ITS2 region) without the need for labeled probes, while maintaining consistent analytical sensitivity and specificity. Despite the limitations of the study, this approach represents a reliable and scalable molecular tool that may complement or support routine diagnosis of leishmaniases, particularly in endemic and resource-limited settings.

## 6. Patents

This study resulted in a patent application to the Brazilian National Institute of Intellectual Property (INPI; https://www.gov.br/inpi/pt-br, access date 24 April 2026; number BR 10 2026 009902 3).

## Figures and Tables

**Table 1:** Characteristics of the oligonucleotide primers designed for the optimization of Leish-qPCR.

Species	Target	Primer 5’–3’	G+C (%)	Amplicon (bp)	Tm (°C)	Reference
*Leishmania* spp.	kDNA	Leish-F GKAGGGGCGTTCTGCG	68.8	116 ^#^	82.3 ± 0.30 ^#^	This study
		Leish-R CCTATWTTACACCAACCCC	47.4			
*L.* (L.) *amazonensis*	ITS2	Lama-F TGGGCTCTCTCTCTCTGTTATG	50.0	218	82.2 ± 0.25	This study
		Lama-R GCCAAACACTCAGGTCTGTAAA	45.5			
*L.* (V.) *braziliensis*	ITS2	Lbra-F GCAGTCTCTCTCTCTCCTCTCT	54.5	249	81.2 ± 0.25	This study
		Lbra-R GTATAGAGAACCACACACACTTGC	45.8			
*Homo sapiens*	*RNaseP*	RNase-F AGATTTGGACCTGCGAGCG	57.9	65	83.1 ± 0.20	[38]
		RNase-R GAGCGGCTGTCTCCACAAGT	60.0			

kDNA: kinetoplast DNA minicircle; ITS2: Internal Transcribed Spacer 2; G+C: guanine and cytosine content; bp: base pair; Tm: melting temperature. ^#^ The amplicon size may vary among species due to sequence variability in the target region.

**Table 2:** Panel of microorganisms used to evaluate the specificity of the Leish-qPCR.

		Targets	
Microorganism	kDNA	LamaITS2	LbraITS2
*Leishmania* (L.) *amazonensis* MHOM/BR/1989/166MJO	+	+	−
*Leishmania* (V.) *braziliensis* MHOM/BR/1987/M11272	+	−	+
*Leishmania* (V.) *equatorensis* IOCL0888	+	−	−
*Leishmania* (V.) *guyanensis* INI565	+	−	−
*Leishmania* (L.) *infantum* MHOM/BR/75/M2903	+	−	−
*Leishmania* (L.) *major* IOCL0581	+	−	−
*Leishmania* (L.) *tropica* IOCL0571	+	−	−
*Trypanosoma cruzi Y*	−	−	−
*Aspergillus flavus* CMRP/UEL	−	−	−
*Aspergillus fumigatus* CMRP/UEL	−	−	−
*Aspergillus niger* CMRP/UEL	−	−	−
*Aspergillus terreus* CMRP/UEL	−	−	−
*Candida albicans* ATCC 26790	−	−	−
*Candidozyma auris* CBS 12766	−	−	−
*Candida glabrata* ATCC 2001	−	−	−
*Candida parapsilosis* ATCC 22019	−	−	−
*Candida tropicalis* ATCC 28707	−	−	−
*Cryptococcus gattii* ATCC 24065	−	−	−
*Cryptococcus neoformans* ATCC 34872	−	−	−
*Paracoccidioides brasiliensis* PB18	−	−	−
*Paracoccidioides lutzii*	−	−	−
*Paracoccidioides brasiliensis* 18	−	−	−
*Klebsiella pneumoniae* ATCC 700603	−	−	−
*Pseudomonas aeruginosa* PAO1	−	−	−
*Enterococcus faecalis* ATCC 29212	−	−	−
*Enterococcus faecium* ATCC 6569	−	−	−
*Staphylococcus aureus* ATCC 13565	−	−	−
*Staphylococcus epidermidis* ATCC 35984	−	−	−
*Staphylococcus haemolyticus* ATCC 29968	−	−	−
*Streptococcus agalactiae* ATCC 13813	−	−	−
*Mycobacterium tuberculosis* H37Rv	−	−	−

IOCL: Coleção de *Leishmania*–CLIOC–do Instituto Oswaldo Cruz/Fiocruz (*Leishmania* Collection–CLIOC–of the Oswaldo Cruz Institute/Fiocruz); INI: Instituto Nacional de Infectologia/Fiocruz (National Institute of Infectious Diseases/Fiocruz); CMRP/UEL: Coleção de Culturas Microbiológicas da Rede Paranaense (Microbiological Culture Collection of the Paraná Network); ATCC: American Type Culture Collection. (+) Presence; (−) Absence.

**Table 3:** Mean Ct values and intra-assay standard deviations (SD) of dilution series, covering a dynamic range from 10^0^ to 10^6^ copies of plasmid control per reaction, obtained with the Leish-qPCR assay.

Species	Target	Copies of Plasmid Control/Reaction						
		10^6^	10^5^	10^4^	10^3^	10^2^	10^1^	10^0^
*Leishmania* spp.	kDNA	11.39 ± 0.33	14.50 ± 0.32	17.45 ±0.17	21.42 ± 0.56	25.39 ± 0.34	28.27 ± 0.32	29.09 ± 0.67
*L.* (L.) *amazonensis*	ITS2	14.13 ± 0.37	18.17 ± 0.18	21.24 ± 0.13	24.71 ± 0.55	27.48 ± 0.02	29.24 ± 0.13	31.73 ± 0.45
*L.* (V.) *braziliensis*	ITS2	16.21 ± 0.11	20.09 ± 0.08	23.85 ± 0.06	27.02 ± 0.27	29.28 ± 0.45	29.65 ± 0.33	30.64 ± 0.42

**Table 4:** Diagnostic performance of Leish-qPCR compared with histopathology.

	Histopathology Positive ^a^	Histopathology Negative ^a^	Total
Leish-qPCR Positive	14	15	29
Leish-qPCR Negative	0	31	31
Total	14	46	60
			
Sensitivity: 100% (95% CI: 76.8–100%) Specificity: 67.4% (95% CI: 52.0–80.5%) PPV: 48.3% (95% CI: 29.4–67.5%) NPV: 100% (95% CI: 88.8–100%) Accuracy: 75.0% (95% CI: 62.1–85.3%)			

^a^ Reference routine histopathological analysis of skin biopsy specimens collected from patients presenting with granulomatous lesions.

## Data Availability

Data are contained within the article and Supplementary Materials.

## Supplementary Materials

The following supporting information can be downloaded at: https://www.mdpi.com/article/10.3390/diagnostics16111704/s1, Table S1. Diagrammatic alignment of (a) kDNA minicircle region of *Leishmania* spp. (partial) and ITS2 of rRNA locus of (b) *L.* (L.). *amazonensis* and (c) *L.* (V.) *braziliensis*. Alignment was computed using Multiple Sequence Alignment ClustalW algorithm. Table S2: Consensus nucleotide sequences of target regions used in melting curve-based real-time PCR (Leish-qPCR). Table S3. Performance of Leish-qPCR with clinical samples. Figure S1. Melting Curve analysis showing the melting peak (Tm) of RNAse P amplicon using monoplex qPCR assays. Figure S2. Sensitivity of Leish-qPCR assays. kDNA minicircle of (a) *L.* (L.) *amazonensis* and (b) *L.* (V.) *braziliensis*. Amplification plots of 10-fold serial dilutions corresponding to 10^0^–10^5^ parasite equivalent numbers. Standard curves were generated by linear regression analysis of threshold cycle (Ct) versus parasite equivalent numbers. The slope (M), regression coefficient (R), and amplification efficiency of the qPCR assay are shown (a,b). The analyses were performed using the Rotor-Gene Q Series instrument and processed with the Rotor-Gene Q Series Software version 2.1.0.9. based on the Ct values obtained during amplification. Figure S3. Statistical agreement between histopathological diagnosis and the Leish-qPCR based on Cohen’s kappa coefficient. The analysis was implemented in Python version 3.13 using the sklearn. metrics library, ensuring a robust quantitative assessment of diagnostic reliability.

## Abbreviations

The following abbreviations are used in this manuscript:

ATL	American Tegumentary Leishmaniasis
BLAST	Basic Local Alignment Search Tool
CL	Cutaneous Leishmaniasis
Ct	Cycle threshold
DNA	Deoxyribonucleic Acid
FBS	Fetal bovine serum
HRM	High-resolution melting
ITS	Internal Transcribed Spacer
kDNA	Kinetoplast DNA
Leish-qPCR	*Leishmania* Real-time Polymerase Chain Reaction
LoD	Limit of Detection
MCL	Mucocutaneous Leishmaniasis
NTC	Negative Template Control
PCR	Polymerase Chain Reaction
qPCR	Real-time PCR
rRNA	Ribosomal Ribonucleic Acid
tRNA	Transfer RNA
SD	Standard deviation
Tm	Melting temperature
VL	Visceral Leishmaniasis
