# Supplementary material for: Molecular Diagnosis of Leishmaniasis: Development of a qPCR Assay for Genus Detection and Differentiation of Leishmania (L.) amazonensis and Leishmania (V.) braziliensis
Source: Diagnostics (Basel). 2026 Jun 2;16(11):1704. doi: 10.3390/diagnostics16111704 (PMC13256636; doi:10.3390/diagnostics16111704)
Supplement: Supplementary file 1 [file diagnostics-16-01704-s001.zip › diagnostics-4233200-supplementary.pdf]

diagnostics

Article

Molecular Diagnosis of Leishmaniasis: Development of a qPCR Assay for Genus Detection and Differentiation of *Leishmania* (*L.*) *amazonensis* and *Leishmania* (*V.*) *braziliensis*

Guilherme Ferreira Correia [1], Bruna Terci Fernandes [2], Paulo Henrique Guilherme Borges [1], Isabela Madeira de Castro [1], Guilherme Bartolomeu-Gonçalves [3], Thiago França Soares [1], Eloiza Teles Caldart [4], Phileno Pinge-Filho [1,5], Ivete Conchon-Costa [5], Vitor Takashiba [6], Nayara Anitelli Artero [6], Marco Aurélio Fornazieri [6], Wander Rogério Pavanelli [5], Eliandro Reis Tavares [1,3], Lucy Megumi Yamauchi [1,3], Celso Vataru Nakamura [1,7,*] and Sueli Fumie Yamada-Ogatta [1,3,*]

[1] Programa de Pós-Graduação em Microbiologia, Universidade Estadual de Londrina, Londrina CEP 86057-970, Paraná, Brazil; guilhermeferreiracorreia@gmail.com (G.F.C.); paulo.guilhermeph@uel.br (P.H.G.B.); isabela.mcastro@uel.br (I.M.d.C.); tavares.eliandro@uel.br (E.R.T.); lionilmy@uel.br (L.M.Y.).

[2] Curso de Farmácia, Faculdade Dom Bosco, Cornélio Procópio CEP 86300-000, Paraná, Brazil; terci.bruna@gmail.com (B.T.F.).

[3] Laboratório de Biologia Molecular de Microrganismos, Departamento de Microbiologia, Universidade Estadual de Londrina, Londrina CEP 86038-350, Paraná, Brazil; guilherme.bartolomeu@uel.br (G.B.-G.)

[4] Departamento de Medicina Veterinária Preventiva, Universidade Estadual de Londrina, Londrina CEP 86057-970, Paraná, Brazil; eloizacaldart@uel.br (E.T.C.)

[5] Programa de Pós-graduação em Patologia Experimental, Universidade Estadual de Londrina, Londrina CEP 86057-970, Paraná, Brazil; pingefilho@uel.br (P.P.-F.); conchon@uel.br (I.C.-C.); wanderpavanelli@uel.br (W.R.P.)

[6] Departamento de Clínica Médica, Universidade Estadual de Londrina, Londrina, CEP 86038-350, Paraná, Brazil; vitortakashiba@gmail.com (V.T.); nayara.anitelli@uel.br (N.A.A.); marcofornazieri@gmail.com (M.A.F.).

[7] Laboratório de Inovação Tecnológica no Desenvolvimento de Fármacos e Cosméticos, Universidade Estadual de Maringá, Maringá CEP 87020-900, Brazil.

* Correspondence: cvnakamura@gmail.com (C.V.N.); ogatta@uel.br (S.F.Y.-O.); Tel.: +55-(43)-3371-5503

 https://doi.org/10.3390/diagnostics16111704

diagnostics MDPI

Supplementary Table S1. Diagrammatic alignment representation of (**a**) kDNA minicircles region of *Leishmania* spp. (partial) and ITS2 of rRNA locus of (**b**) *L.* (*L.*). *amazonensis* and (**c**) *L.* (*V.*) *braziliensis*. Alignment was computed using Multiple Sequence Alignment ClustalW algorithm.

(a) Diagrammatic alignment representation of forward and reverse primers of kDNA minicircles.

```
             ....|....| ....|....| ....|....| ....|....| ....|....| ....|....| ....|....| ....|....| ....|....|
                5         15         25         35         45         55         65         75         85
KY698819.1   ---------- ---------- ---------- --CGACATGC CTCTGGGTAG GGCGTTCTGCG AAAATCGA TTTTTGGCAT ACAGAAACCC
EU437405.1   --TGGGCAAA AATGGGCAAA AATCCCAAAC TTTTCTGGTC CTCCGGGTAG GGCGTTCTGCG AAAATCGA AAAATGGGT- GCAGAAATCC
Z35276.1 L   AATCCCGAAA AATGGGGGAA AATTCCAAAC TTTTCTGGTC CTTCGGGTAG GGCGTTCTGCG AAATCGGA AAAATGGGT- GCAGAAATCC
KY698819.1   ---------- ---------- --TCGTACTC CCCGACATGC CTCTGGGTAG GGCGTTCTGCG AAAATCGA TTTTTGGCAT ACAGAAACCC
KY698902.1   ---------- ---------- -GGCCCAAAC TTTTCTGCCC CGTGGGGGAG GGCGTTCTGCG GGATCCGG AAAATTGATA GCAGAAACCC
Z11556.1 L   ---------- -AGGGGCAGA AATCGTAGCA TGGAGTAGCC CTCCGGGTAG GGCGTTCTGCG AATTTCGA AAAATCGAT- ACAGAAACCC
LshmkDNA-F   ---------- ---------- ---------- ---------- ------GKAG GGCGTTCTGCG-------- ---------- ----------
LshmkDNA-R   ---------- ---------- ---------- ---------- ---------- ---------- ---------- ---------- ----------
           ....|....| ....|....| ....|....| ....|....| ....|....| ....|....| ....|....| ....|....| ....|....|
               95        105        115        125        135        145        155        165        175
KY698819.1   CGTTCAAAAA ATG-CCCAAT TTTCGTGATT TTTGGCCTCC CCGTGCACAA TTAGGGGTTG GTGTAATATA GG---CGGCG CGTCAC----
EU437405.1   CGTTCAAAAA TTG-GCCAAA AATGTCAAAA ATCGGGTCCG AGGCGGGAAA CTGGGGGTTG GTGTAAAATA GGGCCGGGTG G---------
Z35276.1 L   CGTTCAAAAA TTG-GTCAAA AATGCCAAAA ATCAACTCCG GGGCGGAAAA CTGGGGGTTG GTGTAAAATA GG-CCGGATG GTGGGCGGGG
KY698819.1   CGTTCAAAAA ATG-CCCAAT TTTCGTGATT TTTGGCCTCC CCGTGCACAA TTAGGGGTTG GTGTAATATA GG---CGGCG CGTCACACAG
KY698902.1   CGTTCATAAT TTG-GCCAAA AATCCCGAAA TTCGGCTCGG GCGGCCACAA CTGGGGGTTG GTGTAAAATA GG---GGGCG GCTGCTCTGG
Z11556.1 L   CGTTCAAAAA TTCCAGGGAA AATGCCATTT TTGGCCTTGG GGCGTGCAAA CTGGGGGTTG GTGTAAAATA GG-CCGGGTG G--GTCCTGG
LshmkDNA-F   ---------- ---------- ---------- ---------- ---------- ---------- ---------- ---------- ----------
LshmkDNA-R   ---------- ---------- ---------- ---------- ---------- ---GGGGTTG GTGTAAWATA GG-------- ----------
           ....|....| ....|....| ..
              185        195
```

```
KY698819.1   ---------- ---------- --
EU437405.1   ---------- ---------- --
Z35276.1 L   AAATTGGCTG GAATTGGACT TC
KY698819.1   TTTTGGGCTA GCTC------ --
KY698902.1   CCTTT---TA AATTTGGT-- --
Z11556.1 L   AATTC----- ---------- --
LshmkDNA-F   ---------- ---------- --
LshmkDNA-R   ---------- ---------- --
```

(b) Diagrammatic alignment representation of forward and reverse primers of *Leishmania* (*L.*) *amazonensis* ITS2 region (partial) of rDNA locus.

```
                           ....|....| ....|....| ....|....| ....|....| ....|....| ....|....| ....|....| ....|....| ....|..
..|
               5         15         25         35         45         55         65         75         85
MT940889.1   GTCGTAGGTG AACCTGCAGC AGCTGGATCA TTTTCCGATG ATTACACCAA AAAAACATAT ACAAAACTCT CGGGGAGACC TATTCTTTCG
MT940878.1   GTCGTAGGTG AACCTGCAGC AGCTGGATCA TTTTCCGATG ATTACACCAA AAAAACATAT ACAAAACTCT CGGGGAGACC TATTCTTTCG
MT523027.1   GTCGTAGGTG AACCTGCAGC AGCTGGATCA TTTTCCGATG ATTACACCAA AAAAACATAT ACAAAACTCT CGGGGAGACC TATTCTTTCG
AF339753.1   ---GTAGGTG AACCTGCAGC AGCTGGATCA TTTTCCGATG ATTACACCAA AAAAACATAT ACAAAACTCT CGGGGAGACC TATTCTTTCG
MT940882.1   GTCGTAGGTG AACCTGCAGC AGCTGGATCA TTTTCCGATG ATTACACCAA AAAAACATAT ACAAAACTCT CGGGGAGACC TATTCTTTCG
MT940881.1   GTCGTAGGTG AACCTGCAGC AGCTGGATCA TTTTCCGATG ATTACACCAA AAAAACATAT ACAAAACTCT CGGGGAGACC TATTCTTTCG
AJ000314.1   ---------- ---------- --CTGGATCA TTTTCCGATG ATTACACCAA AAAAACATAT ACAAAACTCT CGGGGAGACC TATTCTTTCG
AF339753.1   ---------- ---------- ---------- ---------- ---------- ---------- ---------- ---------- ----------
Lama-F      ---------- ---------- ---------- ---------- ---------- ---------- ---------- ---------- ----------
Rama-R      ---------- ---------- ---------- ---------- ---------- ---------- ---------- ---------- ----------
            ....|....| ....|....| ....|....| ....|....| ....|....| ....|....| ....|....| ....|....| ....|....|
               95        105        115        125        135        145        155        165        175
MT940889.1   ATAGGCGCCT TTCCCACACA TACACAGCAA AGTTTTTGTA CTCAAAAACA ACATTTGCAG TAAACAAAAA ATGGCCGATC GACGTTATAG
MT940878.1   ATAGGCGCCT TTCCCACACA TACACAGCAA AGTTTTTGTA CTCAAAAACA ACATTTGCAG TAAACAAAAA ATGGCCGATC GACGTTATAG
MT523027.1   ATAGGCGCCT TTCCCACACA TACACAGCAA AGTTTTTGTA CTCAAAAACA ACATTTGCAG TAAACAAAAA ATGGCCGATC GACGTTATAG
```

```
AF339753.1   ATAGGCGCCT TTCCCACACA TACACAGCAA AGTTTTTGTA CTCAAAAACA ACATTTGCAG TAAACAAAAA ATGGCCGATC GACGTTATAG
MT940882.1   GTAGGCGCCT TTCCCACACA TACACAGCAA AGTTTTTGTA CTCAAAAACA ACATTTGCAG TAAACAAAAA ATGGCCGATC GACGTTATAG
MT940881.1   ATAGGCGCCT TTCCCACACA TACACAGCAA AGTTTTTGTA CTCAAAAACA ACATTTGCAG TAAACAAAAA ATGGCCGATC GACGTTATAG
AJ000314.1   ATAGGCGCCT TTCCCACACA TACACAGCAA AGTTTTTGTA CTCAAAAACA ACATTTGCAG TAAACAAAAA ATGGCCGATC GACGTTATAG
AF339753.1   ---------- ---------- ---------- ---------- ---------- ---------- ---------- ---------- ----------
Lama-F      ---------- ---------- ---------- ---------- ---------- ---------- ---------- ---------- ----------
Rama-R      ---------- ---------- ---------- ---------- ---------- ---------- ---------- ---------- ----------
            ....|....| ....|....| ....|....| ....|....| ....|....| ....|....| ....|....| ....|....| ....|....|
               185        195        205        215        225        235        245        255        265
MT940889.1   CGCACAACCG CGTATATACA AAAGCAGAGA AAAATGCCCG TTTCAATACG GCGTTTTCCG TTTTTGGGGC GGGGGGGGTG CGTGTGTGGA
MT940878.1   CGCACAACCG CGTATATACA AAAGCAGAGA AAAATGCCCG TTTCAATACG GCGTTTTCCG TTTTTGGGGC GGGGGGG-TG CGTGTGTGGA
MT523027.1   CGCACAACCG CGTATATACA AAAGCAGAGA AAAATGCCCG TTTCAATACG GCGTTTTCCG TTTTTGGGGC GGGGGGG-TG CGTGTGTGGA
AF339753.1   CGCACAACCG CGTATATACA AAAGCAGAGA AAAATGCCCG TTTCAATACG GCGTTTTCCG TTTTTGGGGC GGGGGGT--G CGTGTGTGGA
MT940882.1   CGCACAACCG CGTATATACA AAAGCAGAGA AAAATGCCCG TTTCAATACG GCGTTTTCCG TTTTTGGGGC GGGGGGG-TG CGTGTGTGGA
MT940881.1   CGCACAACCG CGTATATACA AAAGCAGAGA AAAATGCCCG TTTCAATACG GCGTTTTCCG TTTTTGGGGC GGGGGGG-TG CGTGTGTGGA
AJ000314.1   CGCACAACCG CGTATATACA AAAGCAGAGA AAAATGCCCG TTTCAATACG GCGTTTTCCG TTTTTGGGGC GGGGGGG-TG CGTGTGTGGA
AF339753.1   ---------- ---------- ---------- ---------- ---------- ---------- ---------- ---------- ----------
Lama-F      ---------- ---------- ---------- ---------- ---------- ---------- ---------- ---------- ----------
Rama-R      ---------- ---------- ---------- ---------- ---------- ---------- ---------- ---------- ----------
            ....|....| ....|....| ....|....| ....|....| ....|....| ....|....| ....|....| ....|....| ....|....|
               275        285        295        305        315        325        335        345        355
MT940889.1   TAACGGCTCA CATAACGTGT CGCGATGGAT GACTTGGCTT CCTATTTCGT TGAAGAACGC AGTAAAGTGC GATAAGTGGT ATCAATTGCA
MT940878.1   TAACGGCTCA CATAACGTGT CGCGATGGAT GACTTGGCTT CCTATTTCGT TGAAGAACGC AGTAAAGTGC GATAAGTGGT ATCAATTGCA
MT523027.1   TAACGGCTCA CATAACGTGT CGCGATGGAT GACTTGGCTT CCTATTTCGT TGAAGAACGC AGTAAAGTGC GATAAGTGGT ATCAATTGCA
AF339753.1   TAACGGCTCA CATAACGTGT CGCGATGGAT GACTTGGCTT CCTATTTCGT TGAAGAACGC AGTAAAGTGC GATAAGTGGT ATCAATTGCA
MT940882.1   TAACGGCTCA CATAACGTGT CGCGATGGAT GACTTGGCTT CCTATTTCGT TGAAGAACGC AGTAAAGTGC GATAAGTGGT ATCAATTGCA
MT940881.1   TAACGGCTCA CATAACGTGT CGCGATGGAT GACTTGGCTT CCTATTTCGT TGAAGAACGC AGTAAAGTGC GATAAGTGGT ATCAATTGCA
AJ000314.1   TAACGGCTCA CATAACGTGT CGCGATGGAT GACTTGGCTT CCTATTTCGT TGAAGAACGC AGTAAAGTGC GATAAGTGGT ATCAATTGCA
AF339753.1   ---------- ---------- ---------- ---------- ---------- ---------- ---------- ---------- ----------
```

```
Lama-F       ---------- ---------- ---------- ---------- ---------- ---------- ---------- ---------- ----------
Rama-R       ---------- ---------- ---------- ---------- ---------- ---------- ---------- ---------- ----------
             ....|....| ....|....| ....|....| ....|....| ....|....| ....|....| ....|....| ....|....| ....|....|
                 365        375        385        395        405        415        425        435        445
MT940889.1   GAATCATTCA ATTACCGAAT CTTTGAACGC AAACGGCGCA TGGGAGAAGC TCTATTGTGT CATCCCCGTG CATGCCATAT TCTCAGTGTC
MT940878.1   GAATCATTCA ATTACCGAAT CTTTGAACGC AAACGGCGCA TGGGAGAAGC TCTATTGTGT CATCCCCGTG CATGCCATAT TCTCAGTGTC
MT523027.1   GAATCATTCA ATTACCGAAT CTTTGAACGC AAACGGCGCA TGGGAGAAGC TCTATTGTGT CATCCCCGTG CATGCCATAT TCTCAGTGTC
AF339753.1   GAATCATTCA ATTACCGAAT CTTTGAACGC AAACGGCGCA TGGGAGAAGC TCTATTGTGT CATCCCCGTG CATGCCATAT TCTCAGTGTC
MT940882.1   GAATCATTCA ATTACCGAAT CTTTGAACGC AAACGGCGCA TGGGAGAAGC TCTATTGTGT CATCCCCGTG CATGCCATAT TCTCAGTGTC
MT940881.1   GAATCATTCA ATTACCGAAT CTTTGAACGC AAACGGCGCA TGGGAGAAGC TCTATTGTGT CATCCCCGTG CATGCCATAT TCTCAGTGTC
AJ000314.1   GAATCATTCA ATTACCGAAT CTTTGAACGC AAACGGCGCA TGGGAGAAGC TCTATTGTGT CATCCCCGTG CATGCCATAT TCTCAGTGTC
AF339753.1   ---------- ---------- ---------- ---------- ---------- ---------- ---------- ---------- ----------
Lama-F       ---------- ---------- ---------- ---------- ---------- ---------- ---------- ---------- ----------
Rama-R       ---------- ---------- ---------- ---------- ---------- ---------- ---------- ---------- ----------
             ....|....| ....|....| ....|....| ....|....| ....|....| ....|....| ....|....| ....|....| ....|....|
                 455        465        475        485        495        505        515        525        535
MT940889.1   GAACAAAAAA CAACACGCCG CCTCCTCTCT TCTCTATATG TATATATATA CATTATATAT A--TGTGTCT GTGGAAGCCA AGAGGAGGCG
MT940878.1   GAACAAAAAA CAACACGCCG CCTCCTCTCT TCTCTATATG TATATATATA CATTATATAT ATATGTGTCT GTGGAAGCCA AGAGGAGGCG
MT523027.1   GAACAAAAAA CAACACGCCG CCTCCTCTCT TCTCTATATG TATATATATA CATTATATAT ATATGTGTCT GTGGAAGCCA AGAGGAGGCG
AF339753.1   GAACAAAAAA CAACACGCCG CCTCCTCTCT TCTCTATATG TATATATATA CATTATATAT ATATGTGTCT GTGGAAGCCA AGAGGAGGCG
MT940882.1   GAACAAAAAA CAACACGCCG CCTCCTCTCT TCTCTATATG TATATATATA CATTATATAT ATATGTGTCT GTGGAAGCCA AGAGGAGGCG
MT940881.1   GAACAAAAAA CAACACGCCG CCTCCTCTCT TCTCTATATG TATATATATA CATTATATAT ATATGTGTCT GTGGAAGCCA AGAGGAGGCG
AJ000314.1   GAACAAAAAA CAACACGCCG CCTCCTCTCT TCTCTATATG TATATATATA CATTATATAT ATATGTGTCT GTGGAAGCCA AGAGGAGGCG
AF339753.1   --ACAAAAAA CAACACGCCG CCTCCTCTCT TCTCTATATG TATATATATA CATTATATAT ATATGTGTCT GTGGAAGCCA AGAGGAGGCG
Lama-F       ---------- ---------- ---------- ---------- ---------- ---------- ---------- ---------- ----------
Rama-R       ---------- ---------- ---------- ---------- ---------- ---------- ---------- ---------- ----------
             ....|....| ....|....| ....|....| ....|....| ....|....| ....|....| ....|....| ....|....| ....|....|
                 545        555        565        575        585        595        605        615        625
MT940889.1   TGTGTTTGTG TTGTGCACAT ATATATGATA TATATTGTGT TGTGTGCACA CGTAGACAAG TTAGAGTTGG ACAAATACAC ACATGTGCAC
```

```
MT940878.1   TGTGTTTGTG TTGTGCACAT ATATATGATA TATATTGTGT TGTGTGCACA CGTAGACAAG TTAGAGTTGG ACAAATACAC ACATGTGCAC
MT523027.1   TGTGTTTGTG TTGTGCACAT ATATATGATA TATATTGTGT TGTGTGCACA CGTAGACAAG TTAGAGTTGG ACAAATACAC ACATGTGCAC
AF339753.1   TGTGTTTGTG TTGTGCACAT ATATATGATA TATATTGTGT TGTGTGCACA CGTAGACAAG TTAGAGTTGG ACAAATACAC ACATGTGCAC
MT940882.1   TGTGTTTGTG TTGTGCACAT ATATATGATA TATATTGTGT TGTGTGCACA CGTAGACAAG TTAGAGTTGG ACAAATACAC ACATGTGCAC
MT940881.1   TGTGTTTGTG TTGTGCACAT ATATATGATA TATATTGTGT TGTGTGCACA CGTAGACAAG TTAGAGTTGG ACAAATACAC ACATGTGCAC
AJ000314.1   TGTGTTTGTG TTGTGCACAT ATATATGATA TATATTGTGT TGTGTGCACA CGTAGACAAG TTAGAGTTGG ACAAATACAC ACATGTGCAC
AF339753.1   TGTGTTTGTG TTGTGCACAT ATATATGATA TATATTGTGT TGTGTGCACA CGTAGACAAG TTAGAGTTGG ACAAATACAC ACATGTGCAC
Lama-F       ---------- ---------- ---------- ---------- ---------- ---------- ---------- ---------- ----------
Rama-R       ---------- ---------- ---------- ---------- ---------- ---------- ---------- ---------- ----------
            ....|....| ....|....| ....|....| ....|....| ....|....| ....|....| ....|....| ....|....| ....|....|
              635       645       655       665       675       685       695       705       715
MT940889.1   TCTCCTTTGT GTGGGTGCGC GCGTGGAAAA ACTCCTCTCT GGTGCTTGCA AAGCAGTCTC TTTCTCTTCC TCTCTTCCTC TTTCTCTCTC
MT940878.1   TCTCCTTTGT GTGGGTGCGC GCGTGGAAAA ACTCCTCTCT GGTGCTTGCA AAGCAGTCTC TTTCTCTTCC TCTCTTCCTC TTTCTCTCTC
MT523027.1   TCTCCTTTGT GTGGGTGCGC GCGTGGAAAA ACTCCTCTCT GGTGCTTGCA AAGCAGTCTC TTTCTCTTCC TCTCTTCCTC TTTCTCTCTC
AF339753.1   TCTCCTTTGT GTGGGTGCGC GCGTGGAAAA ACTCCTCTCT GGTGCTTGCA AAGCAGTCTC TTTCTCTTCC TCTCTTCCTC TTTCTCTCTC
MT940882.1   TCTCCTTTGT GTGGGTGCGC GCGTGGAAAA ACTCCTCTCT GGTGCTTGCA AAGCAGTCTC TTTCTCTTCC TCTCTTCCTC TTTCTCTCTC
MT940881.1   TCTCCTTTGT GTGGGTGCGC GCGTGGAAAA ACTCCTCTCT GGTGCTTGCA AAGCAGTCTC TTTCTCTTCC TCTCTTCCTC TTTCTCTCTC
AJ000314.1   TCTCCTTTGT GTGGGTGCGC GCGTGGAAAA ACTCCTCTCT GGTGCTTGCA AAGCAGTCTC TTTCTCTTCC TCTCTTCCTC TTTCTCTCTC
AF339753.1   TCTCCTTTGT GTGGGTGCGC GCGTGGAAAA ACTCCTCTCT GGTGCTTGCA AAGCAGTCTC TTTCTCTTCC TCTCTTCCTC TTTCTCTCTC
Lama-F       ---------- ---------- ---------- ---------- ---------- ---------- ---------- ---------- ----------
Rama-R       ---------- ---------- ---------- ---------- ---------- ---------- ---------- ---------- ----------
            ....|....| ....|....| ....|....| ....|....| ....|....| ....|....| ....|....| ....|....| ....|....|
              725       735       745       755       765       775       785       795       805
MT940889.1   TTTTTTCTCC ATTCTCTCCT CTCTTTTTTC ATCAAAAAGA GGGGAGCGAA GAGGGGGACA AGCGAGGGAG GAGAGGGGGG GGGCCGAGGG
MT940878.1   TTTTTTCTCC ATTCTCTCCT CTCTTTTTTC ATCAAAAAGA GGGGAGCGAA GAGGGGGACA AGCGAGGGAG GAGAGGGGGG GGGCCGAGGG
MT523027.1   TTTTTTCTCC ATTCTCTCCT CTCTTTTTTC ATCAAAAAGA GGGGAGCGAA GAGGGGGACA AGCGAGGGAG GAGAGGGGGG GGGCCGAGGG
AF339753.1   TTTTTTCTCC ATTCTCTCCT CTCTTTTTTC ATCAAAAAGA GGGGAGCGAA GAGGGGGACA AGCGAGGGAG GAGAGGGGGG GGGCCGAGGG
MT940882.1   TTTTTTCTCC ATTCTCTCCT CTCTTTTTTC ATCAAAAAGA GGGGAGCGAA GAGGGGGACA AGCGAGGGAG GAGAGGGGGG GGGCCGAGGG
MT940881.1   TTTTTTCTCC ATTCTCTCCT CTCTTTTTTC ATCAAA--GA GGGGAGCGAA GAGGGGGACA AGCGAGGGAG GAGAGGGGGG GGGCCGAGGG
```

```
AJ000314.1   TTTTTTCTCC ATTCTCTCCT CTCTTTTTTC ATCAAAAAGA GGGGAGCGAA GAGGGGGACA AGCGAGGGAG GAGAGGGGGG GGGCCGAGGG
AF339753.1   TTTTTTCTCC ATTCTCTCCT CTCTTTTTTC ATCAAAAAGA GGGGAGCGAA GAGGGGGACA AGCGAGGGAG GAGAGGGGGG GGGCCGAGGG
Lama-F       ---------- ---------- ---------- ---------- ---------- ---------- ---------- ---------- ----------
Rama-R       ---------- ---------- ---------- ---------- ---------- ---------- ---------- ---------- ----------
             ....|....| ....|....| ....|....| ....|....| ....|....| ....|....| ....|....| ....|....| ....|....|
                815        825        835        845        855        865        875        885        895
MT940889.1   ATATAGAGAG GCTGTGACCG GGATTATTAA ACAAAAAACC AAAACGAGAA TTCAACTTCG TTGGCCATTT TTTGCTTAAT GGGTGTGTGT
MT940878.1   ATATAGAGAG GCTGTGACCG GGATTATTAA ACAAAAAACC AAAACGAGAA TTCAACTTCG TTGGCCATTT TTTGCTTAAT GGGTGTGTGT
MT523027.1   ATATAGAGAG GCTGTGACCG GGATTATTAA ACAAAAAACC AAAACGAGAA TTCAACTTCG TTGGCCATTT TTTGCTTAAT GGGTGTGTGT
AF339753.1   ATATAGAGAG GCTGTGACCG GGATTATTAA ACAAAAAACC GAAACGAGAA TTCAACTTCG TTGGCCATTT TTTGCTTAAT GGGTGTGTGT
MT940882.1   ATATAGAGAG GCTGTGACCG GGATTATTAA ACAAAAAACC AAAACGAGAA TTCAACTTCG TTGGCCATTT TTTGCTTAAT GGGTGTGTGT
MT940881.1   ATATAGAGAG GCTGTGACCG GGATTATTAA ACAAAAAACC AAAACGAGAA TTCAACTTCG TTGGCCATTT TTTGCTTAAT GGGTGTGTGT
AJ000314.1   ATATAGAGAG GCTGTGACCG GGATTATTAA ACAAAAAACC AAAACGAGAA TTCAACTTCG TTGGCCATTT TTTGCTTAAT GGGTGTGTGT
AF339753.1   ATATAGAGAG GCTGTGACCG GGATTATTAA ACAAAAAACC GAAACGAGAA TTCAACTTCG TTGGCCATTT TTTGCTTAAT GGGTGTGTGT
Lama-F       ---------- ---------- ---------- ---------- ---------- ---------- ---------- ---------- ---------T
Rama-R       ---------- ---------- ---------- ---------- ---------- ---------- ---------- ---------- ----------
             ....|....| ....|....| ....|....| ....|....| ....|....| ....|....| ....|....| ....|....| ....|....|
                905        915        925        935        945        955        965        975        985
MT940889.1   GGGCTCTCTC TCTCTGTTAT GTGTGTGGTA TATACATATT ATATATATTA GAGTAGGTGT GTGTGTGTGT ATGTGTTTTA CACATATATA
MT940878.1   GGGCTCTCTC TCTCTGTTAT GTGTGTGGTA TATACATATT ATATATATTA GAGTAGGTGT GTGTGTGTGT ATGTGTTTTA CACATATATA
MT523027.1   GGGCTCTCTC TCTCTGTTAT GTGTGTGGTA TATACATATT ATATATATTA GAGTAGGTGT GTGTGTGTGT ATGTGTTTTA CACATATATA
AF339753.1   GGGCTCTCTC TCTCTGTTAT GTGTGTGGTA TATACATATT ATATATATTA GAGTAGGTGT GTGTGTGTGT ATGTGTTTTA CACATATATA
MT940882.1   GGGCTCTCTC TCTCTGTTAT GTGTGTGGTA TATACATATT ATATATATTA GAGTAGGTGT GTGTGTG--T ATGTGTTTTA CACATATATA
MT940881.1   GGGCTCTCTC TCTCTGTTAT GTGTGTGGTA TATACATATT ATATATATTA GAGTAGGTGT GTGTGTGTGT ATGTGTTTTA CACATATATA
AJ000314.1   GGGCTCTCTC TCTCTGTTAT GTGTGTGGTA TATACATATT ATATATATTA GAGTAGGTGT GTGTGTGTGT ATGTGTTTTA CACATATATA
AF339753.1   GGGCTCTCTC TCTCTGTTAT GTGTGTGGTA TATACATATT ATATATATTA GAGTAGGTGT GTGTGTGTGT ATGTGTTTTA CACATATATA
Lama-F       GGGCTCTCTC TCTCTGTTAT G--------- ---------- ---------- ---------- ---------- ---------- ----------
Rama-R       ---------- ---------- ---------- ---------- ---------- ---------- ---------- ---------- ----------
             ....|....| ....|....| ....|....| ....|....| ....|....| ....|....| ....|....| ....|....| ....|....|
```

```
                995        1005       1015       1025       1035       1045       1055       1065       1075
MT940889.1   TTATATGCAC CCTCACTCTC TCATGTATAA TATATATACA CACACACGCA GAGAAAAAAG AGAGGGTTCC TCTGTGTGCT CCCCGCGCAC
MT940878.1   TTATATGCAC CCTCACTCTC TCATGTATAA TATATATACA CACACACGCA GAGAAAAAAG AGAGGGTTCC TCTGTGTGCT CCCCGCGCAC
MT523027.1   TTATATGCAC CCTCACTCTC TCATGTATAA TATATATACA CACACACGCA GAGAAAAAAG AGAGGGTTCC TCTGTGTGCT CCCCGCGCAC
AF339753.1   TTATATGCAC CCTCACTCTC TCATGTATAA TATATATACA CACACACGCA GAGAAAAAAG AGAGGGTTCC TCTGTGTGCT CCCCGCGCAC
MT940882.1   TTATATGCAC CCTCACTCTC TCATGTATAA TATATATACA CACACACGCA GAGAAAAAAG AGAGGGTTCC TCTGTGTGCT CCCCGCGCAC
MT940881.1   TTATATGCAC CCTCACTCTC TCATGTATAA TATATATACA CACACACGCA GAGAAAAAAG GGAGGGTTCC TCTGTGTGCT CCCCGCGCAC
AJ000314.1   TTATATGCAC CCTCACTCTC TCATGTATAA TATATATACA CACACACGCA GAGAAAAAAG AGAGGGTTCC TCTGTGTGCT CCCCGCGCAC
AF339753.1   TTATATGCAC CCTCACTCTC TCATGTATAA TATATATACA CACACACGCA GAGAAAAAAG AGAGGGTTCC TCTGTGTGCT CCCCGCGCAC
Lama-F       ---------- ---------- ---------- ---------- ---------- ---------- ---------- ---------- ----------
Rama-R       ---------- ---------- ---------- ---------- ---------- ---------- ---------- ---------- ----------
            ....|....| ....|....| ....|....| ....|....| ....|....
                1085       1095       1105       1115       1125
MT940889.1   CCCCGACAAC CTTTGTTTAC AGACCTGAGT GTTTGGCAGG ACTACCCGC
MT940878.1   CCCCGACAAC CTTTGTTTAC AGACCTGAGT GTTTGGCAGG ACTACCCGC
MT523027.1   CCCCGACAAC CTTTGTTTAC AGACCTGAGT GTTTGGCAGG ACTACCCGC
AF339753.1   CCCCGACAAC CTTTGTTTAC AGACCTGAGT GTTTGGCAGG ACTACCCGC
MT940882.1   CCCCGACAAC CTTTGTTTAC AGACCTGAGT GTTTGGCAGG ACTACCCGC
MT940881.1   CCCCGACAAC CTTTGTTTAC AGACCTGAGT GTTTGGCAGG ACTACCCGC
AJ000314.1   CCCCGACAAC CTTTGTTTAC AGACCTGAGT GTTTGGCAGG ---------
AF339753.1   CCCCGACAAC CTTTGTTTAC AGACCTGAGT GTTTGGCAGG ACTACCCGC
Lama-F       ---------- ---------- ---------- ---------- ---------
Rama-R       ---------- -----TTTAC AGACCTGAGT GTTTGGC -- ---------
```

(c) Diagrammatic alignment representation of forward and reverse primers of *Leishmania* (*V.*) *brasiliensis* ITS2 region (partial) of rDNA locus.

```
            ....|....| ....|....| ....|....| ....|....| ....|....| ....|....| ....|....| ....|....| ....|....|
                5          15         25         35         45         55         65         75         85
```

```
MT497975.1   ACAAAAAACA ACACACGCCG CCTCCTCTCT TCTCTATATA TATGTATATA TA--TATGTG GGAGCCAGAG GAGGCGTGTG TTTGTGTTGT
MT940879.1   ACAAAAAACA ACACACGCCG CCTCCTCTCT TCTCTATATA TATGTATATA TA--TATGTG GGAGCCAGAG GAGGCGTGTG TTTGTGTTGT
MT940876.1   ACAAAAAACA ACACACGCCG CCTCCTCTCT TCTCTATATA TATGTATATA TA--TATGTG GGAGCCAGAG GAGGCGTGTG TTTGTGTTGT
OY748513.1   ACAAAAAACA ACACACGCCG CCTCCTCTCT TCTCTATATA TATGTATATA TA--TATGTG GGAGCCAGAG GAGGCGTGTG TTTGTGTTGT
OY748513.1   ACAAAAAACA ACACACGCCG CCTCCTCTCT TCGGCATATA TATATATATA TA--TATGTG GGAGCCAGAG GAGGCGTGTG TTTGTGTTGT
FJ753378.1   ACAAAAAACA ACACACGCCG CCTCCTCTCT TCTCTATATA TATGTATATA TA--TATGTG GGAGCCAGAG GAGGCGTGTG TTTGTGTTGT
FJ753380.1   ACAAAAAACA ACACACGCCG CCTCCTCTCT TCTCTATATA TATGTATATA TA--TATGTG GGAGCCAGAG GAGGCGTGTG TTTGTGTTGT
FJ753379.1   ACAAAAAACA ACACACGCCG CCTCCTCTCT TCTCTATATA TATGTATATA TA--TATGTG GGAGCCAGAG GAGGCGTGTG TTTGTGTTGT
LS997626.2   ACAAAAAACA ACACACGCCG CCTCCTCTCT TCTCTATATA TATGTATATA TA--TATGTG GGAGCCAGAG GAGGCGTGTG TTTGTGTTGT
LS997626.2   ACAAAAAACA ACACACGCCG CCTCCTCTCT TCTCTATATA TATGTATATA TA--TATGTG GGAGCCAGAG GAGGCGTGTG TTTGTGTTGT
LS997626.2   ACAAAAAACA ACACACGCCG CCTCCTCTCT TCTCTATATA TATGTATATA TA--TATGTG GGAGCCAGAG GAGGCGTGTG TTTGTGTTGT
LS997626.2   ACAAAAAACA ACACACGCCG CCTCCTCTCT TCTCTATATA TATGTATATA TA--TATGTG GGAGCCAGAG GAGGCGTGTG TTTGTGTTGT
LS997626.2   ACAAAAAACA ACACACGCCG CCTCCTCTCT TCTCTATATA TATGTATATA TATATATGTG GGAGCCAGAG GAGGCGTGTG TTTGTGTTGT
LS997626.2   ACAAAAAACA ACACACGCCG CCTCCTCTCT TCTCTATATA TATGTATATA TA--TATGTG GGAGCCAGAG GAGGCGTGTG TTTGTGTTGT
MT940887.1   ACAAAAAACA ACACACGCCG CCTCCTCTCT TCTCTATATA TATGTTTATA TA--TATGTG GGAGCCAGAG GAGGCGTGTG TTTGTGTTGT
FJ753382.1   ACAAAAAACA ACACACGCCG CCTCCTCTCT TCTCTATATA TATGTATATA TA--TATGTG GGAGCCAGAG GAGGCGTGTG TTTGTGTTGT
FJ753377.1   ACAAAAAACA ACACACGCCG CCTCCTCTCT TCTCTATATA TATGTATATA TA--TATGTG GGAGCCAGAG GAGGCGTGTG TTTGTGTTGT
OY748421.1   ACAAAAAACA ACACACGCCG CCTCCTCTCT TCTCTATATA TATGTATATA TA--TATGTG GGAGCCAGAG GAGGCGTGTG TTTGTGTTGT
OY748421.1   ACAAAAAACA ACACACGCCG CCTCCTCTCT TCTCTATATA TATGTATATA TA--TATGTG GGAGCCAGAG GAGGCGTGTG TTTGTGTTGT
OY748421.1   ACAAAAAACA ACACACGCCG CCTCCTCTCT TCTCTATATA TATGTATATA TA----TGTG GGAGCCAGAG GAGGCGTGTG TTTGTGTTGT
FJ753376.1   ACAAAAAACA ACACACGCCG CCTCCTCTCT TCTCTATATA TATGTATATA TA--TATGTG GGAGCCAGAG GAGGCGTGTG TTTGTGTTGT
MW538634.1   ACAAAAAACA ACACACGCCG CCTCCTCTCT TCTCTATATA TATGTATATA TA--TATGTG GGAGCCAGAG GAGGCGTGTG TTTGTGTTGT
MT940883.1   ACAAAAAACA ACACACGCCG CCTCCTCTCT TCTCTATATA TATGTATATA TA--TATGTG GGAGCCAGAG GAGGCGTGTG TTTGTGTTGT
MT497968.1   ACAAAAAACA ACACACGCCG CCTCCTCTCT TCTCTATATA TATGTATATA TA--TATGTG GGAGCCAGAG GAGGCGTGTG TTTGTGTTGT
JQ061322.1   ACAAAAAACA ACACACGCCG CCTCCTCTCT TCTCTATATA TATGTATATA TA--TATGTG GGAGCCAGAG GAGGCGTGTG TTTGTGTTGT
MT940886.1   ACAAAAAACA ACACACGCCG CCTCCTCTCT TCTCTATATA TATGTATATA TA--TATGTG GGAGCCAGAG GAGGCGTGTG TTTGTGTTGT
MT940877.1   ACAAAAAACA ACACACGCCG CCTCCTCTCT TCTCTATATA TATGTATATA TA--TATGTG GGAGCCAGAG GAGGCGTGTG TTTGTGTTGT
MT940888.1   ACAAAAAACA ACACACGCCG CCTCCTCTCT TCTCTATATA TATGTTTATA TA--TATGTG GGAGCCAGAG GAGGCGTGTG TTTGTGTTGT
AJ300484.1   ACAAAAAACA ACACACGCCG CCTCCTCTCT TCTCTATGTA TATGTATATA TA--TATGTG GGAGCCAGAG GAGGCGTGTG TTTGTGTTGT
```

```
MT497927.1   ACAAAAAACA ACACACGCCG CCTCCTCTCT TCTCTATATA TATGTATATA TA--TATGTG GGAGCCAGAG GAGGCGTGTG TTTGTGTTGT
MT497929.1   ACAAAAAACA ACACACGCCG CCTCCTCTCT TCTCTATATA TATGTATATA TA--TATGTG GGAGCCAGAG GAGGCGTGTG TTTGTGTTGT
FJ753383.1   ACAAAAAACA ACACACGCCG CCTCCTCTCT TCTCTATATA TATGTATATA TATATATGTG GGAGCCAGAG GAGGCGTGTG TTTGTGTTGT
MT497941.1   ACAAAAAACA ACACACGCCG CCTCCTCTCT TCTCTATATA TATGTTTATA TA--TATGTG GGAGCCAGAG GAGGCGTGTG TTTGTGTTGT
FJ753384.1   ACAAAAAACA ACACACGCCG CCTCCTCTCT TCTCTATATA TATGTATATA TATATATGTG GGAGCCAGAG GAGGCGTGTG TTTGTGTTGT
MT940880.1   ACAAAAAACA ACACACGCCG CCTCCTCTCT TCTCTATATA TATATGTATA TA--TATGTG GGAGCCAGAG GAGGCGTGTG TTTGTGTTGT
FJ753374.1   ACAAAAAACA ACACACGCCG CCTCCTCTCT TCTCTATATA TATGTATATA TA--TATGTG GGAGCCAGAG GAGGCGTGTG TTTGTGTTGT
FJ753375.1   ACAAAAAACA ACACACGCCG CCTCCTCTCT TCTCTATATA TATGTATATA TATATATGTG GGAGCCAGAG GAGGCGTGTG TTTGTGTTGT
FJ753381.1   ACAAAAAACA ACACACGCCG CCTCCTCTCT TCTCTATATA TATGTATATA TATATATGTG GGAGCCAGAG GAGGCGTGTG TTTGTGTTGT
MT497946.1   ACAAAAAACA ACACACGCCG CCTCCTCTCT TCTCTATATA TAT--GTATA TA--TATGTG GGAGCCAGAG GAGGCGTGTG TTTGTGTTGT
AJ300483.1   ACAAAAAACA ACACACGCCG CCTCCTCTCT TCTCTATATA TATGTATA-A TATATATGTG GGAGCCAGAG GAGGCGTGTG TTTGTGTTGT
FJ753385.1   ACAAAAAACA ACACACGCCG CCTCCTCTCT TCTCTATATA TATGTATATA ----TATGTG GGAGCCAGAG GAGGCGTGTG TTTGTGTTGT
Lbraz-F      ---------- ---------- ---------- ---------- ---------- ---------- ---------- ---------- ----------
Lbraz-R      ---------- ---------- ---------- ---------- ---------- ---------- ---------- ---------- ----------
            ....|....| ....|....| ....|....| ....|....| ....|....| ....|....| ....|....| ....|....| ....|....|
                95        105       115       125       135       145       155       165       175
MT497975.1   GCGCATATAT ATATATATAT A------CGC ACACGTATAC AAGTCAGAGT TGGACAAAAA -TACACACCT GCACTCTCTT TTCGAGATGT
MT940879.1   GCGCATATAT ATATATATAT A------CGC ACACGTATAC AAGTCAGAGT TGGACAAAAA -TACACACCT GCACTCTCTT TTCGAGATGT
MT940876.1   GCGCATATAT ATATATATAT ------ACGC ACACGTATAC AAGTCAGAGT TGGACAAAAA -TACACACCT GCACTCTCTT TTCGAGATGT
OY748513.1   GCGCATATAT ATATATATAT ------ACGC ACACGTATAC AAGTCAGAGT TGGACAAAAA -TACACACCT GCACTCTCTT TTCGAGATGT
OY748513.1   GCGCATATAT ATATATATAT ------ACGC ACACGTATAC AAGTCAGAGT TGGACAAAAA -TACACACCT GCACTCTCTT TTCGAGATGT
FJ753378.1   GCGCATATAT ATATATATAT ------ACGC ACACGTATAC AAGTCAGAGT TGGACAAAAA -TACACACCT GCACTCTCTT TTCGAGATGT
FJ753380.1   GCGCATATAT ATATATATAT AT----ACGC ACACGTATAC AAGTCAGAGT TGGACAAAAA -TACACACCT GCACTCTCTT TTCGAGATGT
FJ753379.1   GCGCATATAT ATATATATAT A------CGC ACACGTATAC AAGTCAGAGT TGGACAAAAA -TACACACCT GCACTCTCTT TTCGAGATGT
LS997626.2   GCGCATATAT ATATATATAT AT----ACGC ACACGTATAC AAGTCAGAGT TGGACAAAAA -TACACACCT GCACTCTCTT TTCGAGATGT
LS997626.2   GCGCATATAT ATATATATAT ATAT--ACGC ACACGTATAC AAGTCAGAGT TGGACAAAAA -TACACACCT GCACTCTCTT TTCGAGATGT
LS997626.2   GCGCATATAT ATATATATAT ATAT--ACGC ACACGTATAC AAGTCAGAGT TGGACAAAAA -TACACACCT GCACTCTCTT TTCGAGATGT
LS997626.2   GCGCATATAT ATATATATAT ATAT--ACGC ACACGTATAC AAGTCAGAGT TGGACAAAAA -TACACACCT GCACTCTCTT TTCGAGATGT
LS997626.2   GCGCATATAT ATATATATAT AT----ACGC ACACGTATAC AAGTCAGAGT TGGACAAAAA ATACACACCT GCACTCTCTT TTCGAGATGT
```

```
LS997626.2   GCGCATATAT ATATATATAT AT----ACGC ACACGTATAC AAGTCAGAGT TGGACAAAAA -TACACACCT GCACTCTCTT TTCGAGATGT
MT940887.1   GCGCATATAT ATATATATAT ------GCGC ACACGTATAC AAGTCAGAGT TGGACAAAAA -TACACACCT GCACTCTCTT TTCGAGATGT
FJ753382.1   GCGCATATAT ATATATATAT ATAT--ACGC ACACGTATAC AAGTCAGAGT TGGACAAAAA -TACACACCT GCACTCTCTT TTCGAGATGT
FJ753377.1   GCGCATATAT ATATATATA- -------CGC ACACGTATAC AAGTCAGAGT TGGACAAAAA -TACACACCT GCACTCTCTT TTCGAGATGT
OY748421.1   GCGCATATAT ATATATATAT ATAT--ACGC ACACGTATAC AAGTCAGAGT TGGACAAAAA -TACACACCT GCACTCTCTT TTCGAGATGT
OY748421.1   GCGCATATAT ATATATATAT ATAT--ACGC ACACGTATAC AAGTCAGAGT TGGACAAAAA -TACACACCT GCACTCTCTT TTCGAGATGT
OY748421.1   GCGCATATAT ATATATATAT ATAT--ACGC ACACGTATAC AAGTCAGAGT TGGACAAAAA -TACACACCT GCACTCTCTT TTCGAGATGT
FJ753376.1   GCGCATATAT ATATATATA- -------CGC ACACGTATAC AAGTCAGAGT TGGACAAAAA -TACACACCT GCACTCTCTT TTCGAGATGT
MW538634.1   GCGCATATAT ATATATATAT ------ACGC ACACGTATAC AAGTCAGAGT TGGACAAAAA -TACACACCT GCACTCTCTT TTCGAGATGT
MT940883.1   GCGCATATAT ATATATATAT ------ACGC ACACGTATAC AAGTCAGAGT TGGACAAAAA -TACACACCT GCACTCTCTT TTCGAGATGT
MT497968.1   GCGCATATAT ATATATATAT ------ACGC ACACGTATAC AAGTCAGAGT TGGACAAAAA -TACACACCT GCACTCTCTT TTCGAGATGT
JQ061322.1   GCGCATATAT ATATATATAT ------ACGC ACACGTATAC AAGTCAGAGT TGGACAAAAA -TACACACCT GCACTCTCTT TTCGAGATGT
MT940886.1   GCGCATATAT ATATATATAT ------ACGC ACACGTATAC AAGTCAGAGT TGGACAAAAA -TACACACCT GCACTCTCTT TTCGAGATGT
MT940877.1   GCGCATATAT ATATATATAT ------ACGC ACACGTATAC AAGTCAGAGT TGGACAAAAA -TACACACCT GCACTCTCTT TTCGAGATGT
MT940888.1   GCGCATATAT ATATATATAT ATAT--GCGC ACACGTATAC AAGTCAGAGT TGGACAAAAA -TACACACCT GCACTCTCTT TTCGAGATGT
AJ300484.1   GCGCATATAT ATATATATAT ------ACGC ACACGTANAC ANGTCAGAGT TGGACAAAAA -TACACACCT GCACTCTCTT TTCGAGATGT
MT497927.1   GCGCATATAT ATATATATAT ATATATACGC ACACGTATAC AAGTCAGAGT TGGACAAAAA -TACACACCT GCACTCTCTT TTCGAGATGT
MT497929.1   GCGCATATAT ATATATAT-- ------ACGC ACACGTATAC AAGTCAGAGT TGGACAAAAA -TACACACCT GCACTCTCTT TTCGAGATGT
FJ753383.1   GCGCATATAT ATATATAT-- ------ACGC ACACGTATAC AAGTCAGAGT TGGACAAAAA -TACACACCT GCACTCTCTT TTCGAGATGT
MT497941.1   GCGCATATAT ATATATA--- -------CGC ACACGTATAC AAGTCAGAGT TGGACAAAAA -TACACACCT GCACTCTCTT TTCGAGATGT
FJ753384.1   GCGCATATAT ATATATAT-- ------ACGC ACACGTATAC AAGTCAGAGT TGGACAAAAA -TACACACCT GCACTCTCTT TTCGAGATGT
MT940880.1   GCGCATATAT ATATATATAT AT----ACGC ACACGTATAC AAGTCAGAGT TGGACAAAAA -TACACACCT GCACTCTCTT TTCGAGATGT
FJ753374.1   GCGCATATAT ATATATAT-- ------ACGC ACACGTATAC AAGTCAGAGT TGGACAAAAA -TACACACCT GCACTCTCTT TTCGAGATGT
FJ753375.1   GCGCATATAT ATATATA--- -------CGC ACACGTATAC AAGTCAGAGT TGGACAAAAA -TACACACCT GCACTCTCTT TTCGAGATGT
FJ753381.1   GCGCATATAT ATATATA--- -------CGC ACACGTATAC AAGTCAGAGT TGGACAAAAA -TACACACCT GCACTCTCTT TTCGAGATGT
MT497946.1   GCGCATATAT ATATATATAT ------ACGC ACACGTATAC AAGTCAGAGT TGGACAAAAA -TACACACCT GCACTCTCTT TTCGAGATGT
AJ300483.1   GCGCATATAT ATATATATAT ------AGAG AGAGAGATAC AAGTCAGAGT TGGACAAAAA -TACACACCT GCACTCTCTT TTCGAGATGT
FJ753385.1   GCGCATATAT ATATATA--- -------CGC ACACGTATAC AAGTCAGAGT TGGACAAAAA -TACACACCT GCACTCTCTT TTCGAGATGT
Lbraz-F      ---------- ---------- ---------- ---------- ---------- ---------- ---------- ---------- ----------
```

```
Lbraz-R      ---------- ---------- ---------- ---------- ---------- ---------- ---------- ---------- ----------
            ....|....| ....|....| ....|....| ....|....| ....|....| ....|....| ....|....| ....|....| ....|....|
                185       195       205       215       225       235       245       255       265
MT497975.1   GCGTGTGGAA AAACTCCTCT CTGGTGGTTG CAAAGCAGTC TCTCTCTCTC CTCTCTCCTC TCTATTCTCT CTTCTCCTCT CACTTTAAGA
MT940879.1   GCGTGTGGAA AAACTCCTCT CTGGTGGTTG CAAAGCAGTC TCTCTCTCTC CTCTCTCCTC TCTATTCTCT CTTCTCCTCT CACTTTAAGA
MT940876.1   GCGTGTGGAA AAACTCCTCT CTGGTGGTTG CAAAGCAGTC TCTCTCTCTC CTCTCTCCTC TTTATTCTCT CTTCTCCTCT CACTTTAAGA
OY748513.1   GCGTGTGGAA AAACTCCTCT CTGGTGGTTG CAAAGCAGTC TCTCTCTCTC CTCTCTCCTC TCTATTCTCT CTTCTCCTCT CACTTTAAGA
OY748513.1   GCGTGTGGAA AAACTCCTCT CTGGTGGTTG CAAAGCAGTC TCTCTCTCTC CTCTCTCCTC TCTATTCTCT CTTCTCCTCT CACTTTAAGA
FJ753378.1   GCGTGTGGAA AAACTCCTCT CTGGTGGTTG CAAAGCAGTC TCTCTCTCTC CTCTCTCCTC TCTATTCTCT CTTCTCCTCT CACTTTAAGA
FJ753380.1   GCGTGTGGAA AAACTCCTCT CTGGTGGTTG CAAAGCAGTC TCTCTCTCTC CTCTCTCCTC TCTATTCTCT CTTCTCCTCT CACTTTAAGA
FJ753379.1   GCGTGTGGAA AAACTCCTCT CTGGTGGTTG CAAAGCAGTC TCTCTCTCTC CTCTCTCCTC TCTATTCTCT CTTCTCCTCT CACTTTAAGA
LS997626.2   GCGTGTGGAA AAACTCCTCT CTGGTGGTTG CAAAGCAGTC TCTCTCTCTC CTCTCTCCTC TCTATTCTCT CTTCTCCTCT CACTTTAAGA
LS997626.2   GCGTGTGGAA AAACTCCTCT CTGGTGGTTG CAAAGCAGTC TCTCTCTCTC CTCTCTCCTC TCTATTCTCT CTTCTCCTCT CACTTTAAGA
LS997626.2   GCGTGTGGAA AAACTCCTCT CTGGTGGTTG CAAAGCAGTC TCTCTCTCTC CTCTCTCCTC TCTATTCTCT CTTCTCCTCT CACTTTAAGA
LS997626.2   GCGTGTGGAA AAACTCCTCT CTGGTGGTTG CAAAGCAGTC TCTCTCTCTC CTCTCTCCTC TCTATTCTCT CTTCTCCTCT CACTTTAAGA
LS997626.2   GCGTGTGGAA AAACTCCTCT CTGGTGGTTG CAAAGCAGTC TCTCTCTCTC CTCTCTCCTC TCTATTCTCT CTTCTCCTCT CACTTTAAGA
LS997626.2   GCGTGTGGAA AAACTCCTCT CTGGTGGTTG CAAAGCAGTC TCTCTCTCTC CTCTCTCCTC TCTATTCTCT CTTCTCCTCT CACTTTAAGA
MT940887.1   GCGTGTGGAA AAACTCCTCT CTGGTGCTTG CAAAGCAGTC TCTCTCTCTC CTCTCTCCTC TCTATTCTCT CTTCTCCTCT CACTTTAAGA
FJ753382.1   GCGTGTGGAA AAACTCCTCT CTGGTGGTTG CAAAGCAGTC TCTCTCTCTC CTCTCTCCTC TCTATTCTCT CTTCTCCTCT CACTTTAAGA
FJ753377.1   GCGTGTGGAA AAACTCCTCT CTGGTGGTTG CAAAGCAGTC TCTCTCTCTC CTCTCTCCTC TCTATTCTCT CTTCTCCTCT CACTTTAAGA
OY748421.1   GCGTGTGGAA AAACTCCTCT CTGGTGGTTG CAAAGCAGTC TCTCTCTCTC CTCTCTCCTC TCTATTCTCT CTTCTCCTCT CACTTTAAGA
OY748421.1   GCGTGTGGAA AAACTCCTCT CTGGTGGTTG CAAAGCAGTC TCTCTCTCTC CTCTCTCCTC TCTATTCTCT CTTCTCCTCT CACTTTAAGA
OY748421.1   GCGTGTGGAA AAACTCCTCT CTGGTGGTTG CAAAGCAGTC TCTCTCTCTC CTCTCTCCTC TCTATTCTCT CT-CTCCTCT CACTTTAAGG
FJ753376.1   GCGTGTGGAA AAACTCCTCT CTGGTGGTTG CAAAGCAGTC TCTCTCTCTC CTCTCTCCTC TCTATTCTCT CTTCTCCTCT CACTTTAAGA
MW538634.1   GCGTGTGGAA AAACTCCTCT CTGGTGGTTG CAAAGCAGTC TCTCTCTCTC CTCTCTCCTC TCTATTCTCT CTTCTCCTCT CACTTTAAGA
MT940883.1   GCGTGTGGAA AAACTCCTCT CTGGTGGTTG CAAAGCAGTC TCTCTCTCTC CTCTCTCCTC TCTATTCTCT CTTCTCCTCT CACTTTAAGA
MT497968.1   GCGTGTGGAA AAACTCCTCT CTGGTGGTTG CAAAGCAGTC TCTCTCTCTC CTCTCTCCTC TCTATTCTCT CTTCTCCTCT CACTTTAAGA
JQ061322.1   GCGTGTGGAA AAACTCCTCT CTGGTGGTTG CAAAGCAGTC TCTCTCTCTC CTCTCTCCCC TCTATTCTCT CTTCTCCTCT CACTTTAAGA
MT940886.1   GCGTGTGGAA AAACTCCTCT CTGGTGGTTG CAAAGCAGTC TCTCTCTCTC CTCTCTCCTC TCTATTCTCT CTTCTCCTCT CACTTTAAGA
```

```
MT940877.1   GCGTGTGGAA AAACTCCTCT CTGGTGGTTG CAAAGCAGTC TCTCTCTCTC CTCTCTCCTC TCTATTCTCT CTTCTCCTCT CACTTTAAGA
MT940888.1   GCGTGTGGAA AAACTCCTCT CTGGTGCTTG CAAAGCAGTC TCTCTCTCTC CTCTCTCCTC TCTATTCTCT CTTCTCCTCT CACTTTAAGA
AJ300484.1   GCGTGTGGAA AAACTCCTCT CTGGTGGTTG CAAAGCAGTC TCTCTCTCTC CTCTCTCCTC TCTATTCTCT CTTCTCCTCT CACTTTAAGA
MT497927.1   GCGTGTGGAA AAACTCCTCT CTGGTGGTTG CAAAGCAGTC TCTCTCTCTC CTCTCTCCTC TCTATTCTCT CTTCTCCTCT CACTTTAAGA
MT497929.1   GCGTGTGGAA AAACTCCTCT CTGGTGGTTG CAAAGCAGTC TCTCTCTCTC CTCTCTCCTC TCTATTCTCT CTTCTCCTCT CACTTTAAGA
FJ753383.1   GCGTGTGGAA AAACTCCTCT CTGGTGGTTG CAAAGCAGTC TCTCTCTCTC CTCTCTCCTC TCTATTCTCT CTTCTCCTCT CACTTTAAGA
MT497941.1   GCGTGTGGAA AAACTCCTCT CTGGTGCTTG CAAAGCAGTC TCTCTCTCTC CTCTCTCCTC TCTATTCTCT CTTCTCCTCT CACTTTAAGA
FJ753384.1   GCGTGTGGAA AAACTCCTCT CTGGTGGTTG CAAAGCAGTC TCTCTCTCTC CTCTCTCCTC TCTATTCTCT CTTCTCCTCT CACTTTAAGA
MT940880.1   GCGTGTGGAA AAACTCCTCT CTGGTGGTTG CAAAGCAGTC TCTCTCTCTC CTCTCTCCTC TCTATTCTCT CTTCTCCTCT CACTTTAAGA
FJ753374.1   GCGTGTGGAA AAACTCCTCT CTGGTGGTTG CAAAGCAGTC TCTCTCTCTC CTCTCTCCTC TCTATTCTCT CTTCTC---T CACTTTAAGA
FJ753375.1   GCGTGTGGAA AAACTCCTCT CTGGTGGTTG CAAAGCAGTC TCTCTCTCTC CTCTCTCCTC TCTATTCTCT CTTCTCCTCT CACTTTAAGA
FJ753381.1   GCGTGTGGAA AAACTCCTCT CTGGTGGTTG CAAAGCAGTC TCTCTCTCTC CTCTCTCCTC TCTATTCTCT CTTCTCCTCT CACTTTAAGA
MT497946.1   GCGTGTGGAA AAACTCCTCT CTGGTGGTTG CAAAGCAGTC TCTCTCTCTC CTCTCTCCTC TCTATTCTCT CTTCTCCTCT CACTTTAAGA
AJ300483.1   GCGTGTGGAA AAACTCCTCT CTGGTGGTTG CAAAGCAGTC TCTCTCTCTC CTCTCTCCTC TCTATTCTCT CTTCTCCTCT CACTTTAAGA
FJ753385.1   GCGTGTGGAA AAACTCCTCT CTGGTGGTTG CAAAGCAGTC TCTCTCTCTC CTCTCTCCTC TCTATTCTCT CTTCTC---T CACTTTAAGA
Lbraz-F      ---------- ---------- ---------- ----GCAGTC TCTCTCTCTC CTCTCT---- ---------- ---------- ----------
Lbraz-R      ---------- ---------- ---------- ---------- ---------- ---------- ---------- ---------- ----------
             ....|....| ....|....| ....|....| ....|....| ....|....| ....|....| ....|....| ....|....| ....|....|
                275        285        295        305        315        325        335        345        355
MT497975.1   GGGGG-GAGA GGAGGGGTTT TATATAGAGA AGAGGGGTGT GGGGGGGG-- -GAGGCTGTG ACCAGGATAA TTAAACAAAA AACCAAAACG
MT940879.1   GGGGG-GAGA GGAGGGGTTT TATATAGAGA AGAGGGGTGT GGGGGGGG-- -GAGGCTGTG ACCAGGATAA TTAAACAAAA AACCAAAACG
MT940876.1   GGGGG-GAGA GGAGGGGTTT TATATAGAGA AGAGGGGTGT GGGGGGG--- GGAGGCTGTG ACCAGGATAA TTAAACAAAA AACCAAAACG
OY748513.1   GGGGG-GAGA GGAGGGGTTT TATATAGAGA AGAGGGGTGT GGGGGGG--- -GAGGCTGTG ACCAGGATAA TTAAACAAAA AACCAAAACG
OY748513.1   GGGGG-GAGA GGAGGGGTTT TATATAGAGA AGAGGGGTGT GGGGGGG--- G-AGGCTGTG ACCAGGATAA TTAAACAAAA AACCAAAACG
FJ753378.1   GGGGG-GAGA GGAGGGGTTT TATATAGAGA AGAGGGGTGT GGGGGGG--- -GAGGCTGTG ACCAGGATAA TTAAACAAAA AACCAAAACG
FJ753380.1   GGGGG-GAGA GGAGGGGTTT TATATAGAGA AGAGGGGTGT GGGGGGGG-- -GAGGCTGTG ACCAGGATAA TTAAACAAAA AACCAAAACG
FJ753379.1   GGGGG-GAGA GGAGGGGTTT TATATAGAGA AGAGGGGTGT GGGGGGGG-- --AGGCTGTG ACCAGGATAA TTAAACAAAA AACCAAAACG
LS997626.2   GGGGG-GAGA GGAGGGGTTT TATATAGAGA AGAGGGGTGT GGGGGGGG-- GGAGGCTGTG ACCAGGATAA TTAAACAAAA AACCAAAACG
LS997626.2   GGGGG-GAGA GGAGGGGTTT TATATAGAGA AGAGGGGTGT GGGGGGGG-- -GAGGCTGTG ACCAGGATAA TTAAACAAAA AACCAAAACG
```

LS997626.2	GGGGG-GAGA GGAGGGGTTT TATATAGAGA AGAGGGGTGT GGGGGGGGG- -GAGGCTGTG ACCAGGATAA TTAAACAAAA AACCAAAACG	375
LS997626.2	GGGGGGGAGA GGAGGGGTTT TATATAGAGA AGAGGGGTGT GGGGGGGGG- -GAGGCTGTG ACCAGGATAA TTAAACAAAA AACCAAAACG	376
LS997626.2	GGGGG-GAGA GGAGGGGTTT TATATAGAGA AGAGGGGTGT GGGGGGGGGG GGAGGCTGTG ACCAGGATAA TTAAACAAAA AACCAAAACG	377
LS997626.2	GGGG--GAGA GGAGGGGTTT TATATAGAGA AGAGGGGTGT GGGGGG---- -GAGGCTGTG ACCAGGATAA TTAAACAAAA AACCAAAACG	378
MT940887.1	GGGGG-GAGA GGAGGGGTTT TATATAGAGA AGAGGGGTGT GGGGGGGG-- G-AGGCTGTG ACCAGGATAA TTAAACAAAA AACCAAAACG	379
FJ753382.1	GGGGG-GAGA GGAGGGGTTT TATATAGAGA AGAGGGGTGT GGGGGGGG-- -GAGGCTGTG ACCAGGATAA TTAAACAAAA AACCAAAACG	380
FJ753377.1	GGGGG-GAGA GGAGGGGTTT TATATAGAGA AGAGGGGTGT GGGGGGG--- -GAGGCTGTG ACCAGGATAA TTAAACAAAA AACCAAAACG	381
OY748421.1	GGGGG-GAGA GGAGGGGTTT TATATAGAGA AGAGGGGTGT GGGGGGGGG- -GAGGCTGTG ACCAGGATAA TTAAACAAAA AACCAAAACG	382
OY748421.1	GGGGG-GAGA GGAGGGGTTT TATATAGAGA AGAGGGGTGT GGGGGGGGG- -GAGGCTGTG ACCAGGATAA TTAAACAAAA AACCAAAACG	383
OY748421.1	GGGGG-GGGG GGGGGGGTTT TATATAGAGA AGGGGGGGGG GGGGGGGG-- GGAGGCTGTG ACCAGGATAA TTAAACAAAA AACCAAAACG	384
FJ753376.1	GGGGG-GAGA GGAGGGGTTT TATATAGAGA AGAGGGGTGT GGGGGGGG-- --AGGCTGTG ACCAGGATAA TTAAACAAAA AACCAAAACG	385
MW538634.1	GGGGG-GAGA GGAGGGGTTT TATATAGAGA AGAGGGGTGT GGGGGGGGG- GGAGGCTGTG ACCAGGATAA TTAAACAAAA AACCAAAACG	386
MT940883.1	GGGGG-GAGA GGAGGGGTTT TATATAGAGA AGAGGGGTGT GGGGGGGGG- GGAGGCTGTG ACCAGGATAA TTAAACAAAA AACCAAAACG	387
MT497968.1	GGGGG-GAGA GGAGGGGTTT TATATAGAGA AGAGGGGTGT GGGGGGG--- GGAGGCTGTG ACCAGGATAA TTAAACAAAA AACCAAAACG	388
JQ061322.1	GGGGG-GAGA GGAGGGGTTT TATATAGAGA AGAGGGGTGT GGGGGGGG-- GGAGGCTGTG ACCAGGATAA TTAAACAAAA AACCAAAACG	389
MT940886.1	GGGGG-GAGA GGAGGGGTTT TATATAGAGA AGAGGGGTGT GGGGGGGGGG GGAGGCTGTG ACCAGGATAA TTAAACAAAA AACCAAAACG	390
MT940877.1	GGGGG-GAGA GGAGGGGTTT TATATAGAGA AGAGGGGTGT GGGGGGGGG- GGAGGCTGTG ACCAGGATAA TTAAACAAAA AACCAAAACG	391
MT940888.1	GGGGG-GAGA GGAGGGGTTT TATATAGAGA AGAGGGGTGT GGGGGGGG-- GGAGGCTGTG ACCAGGATAA TTAAACAAAA AACCAAAACG	392
AJ300484.1	GGGGG-GAGA GGAGGGGTTT TATATAGAGA AGAGGGGTGT GTGGGGG--- GGAGGCTGTG ACCAGGATAA TTAAACAAAA AACCAAAACG	393
MT497927.1	GGGGG-GAGA GGAGGGGTTT TATATAGAGA AGAGGGGTGT GGGGGGGG-- -GAGGCTGTG ACCAGGATAA TTAAACAAAA AACCAAAACG	394
MT497929.1	GGGGG-GAGA GGAGGGGTTT TATATAGAGA AGAGGGGTGT GGGGGGG--- GGAGGCTGTG ACCAGGATAA TTAAACAAAA AACCAAAACG	395
FJ753383.1	GGGGG-GAGA GGAGGGGTTT TATATAGAGA AGAGGGGTGT GGGGGGGA-- GGAGGCTGTG ACCAGGATAA TTAAACAAAA AACCAAAACG	396
MT497941.1	GGGGG-GAGA GGAGGGGTTT TATATAGAGA AGAGGGGTGT GGGGGGGG-- G-AGGCTGTG ACCAGGATAA TTAAACAAAA AACCAAAACG	397
FJ753384.1	GGGGG-GAGA GGAGGGGTTT TATATAGAGA AGAGGGGTGT GGGGGGG--- GGAGGCTGTG ACCAGGATAA TTAAACAAAA A-CCAAAACG	398
MT940880.1	GGGGG-GAGA GGAGGGGTTT TATATAGAGA AGAGGGGTGT GGGGGGG--- G-AGGCTGTG ACCAGGATAA TTAAACAAAA AACCAAAACG	399
FJ753374.1	GGGGG-GAGA GGAGGGGTTT TATATAGAGA AGAGGGGTGT GGGGGGG--- GGAGGCTGTG ACCAGGATAA TTAAACAAAA AACCAAAACG	400
FJ753375.1	GGGGG-GAGA GGAGGGGTTT TATATAGAGA AGAGGGGTGT GGGGGGG--- GGAGGCTGTG ACCAGGATAA TTAAACAAAA AACCAAAACG	401
FJ753381.1	GGGGA-GAGA GGAGGGGTTT TATATAGAGA AGAGGGGTGT GGGGGGG--- GGAGGCTGTG ACCAGGATAA TTAAACAAAA AACCAAAACG	402
MT497946.1	GGGGG-GAGA GGAAGGGTTT TATATAGAGA AGAGGGGTGT GGGGGGG--- GGAGGCTGTG ACCAGGATAA TTAAACAAAA AACCAAAACG	403

```
AJ300483.1  GGGGG-GAGA GGAGGGGTTT TATATAGAGA AGAGGGGTGT GGGGGGGG-- -AGGACTGTG ACCAGGATAA TTAAACAAAA AACCAAAACG
FJ753385.1  GAGGG-GAGA GGAGGGGTTT TATATAGAGA AGAGGGGTGT GGGGGGG--- GGAGGCTGTG ACCAGGATAA TTAAACAAAA AACCAAAACG
Lbraz-F     ---------- ---------- ---------- ---------- ---------- ---------- ---------- ---------- ----------
Lbraz-R     ---------- ---------- ---------- ---------- ---------- ---------- ---------- ---------- ----------
           ....|....| ....|....| ....|....| ....|....| ....|....| ....|....| ....|....| ....|....| ....|....|
              365       375       385       395       405       415       425       435       445
MT497975.1  AGAATTCAAC TTCGC-GTTG GC--CATTTT TTGCTTAATG GTGTGGGACC TCCTTTCTCT TTCTCTCTCT CTCCGTGTAT ATGCAAGTGT
MT940879.1  AGAATTCAAC TTCGC-GTTG GC--CATTTT TTGCTTAATG GTGTGGGACC TCCTTTCTCT TTCTCTCTCT CTCCGTGTAT ATGCAAGTGT
MT940876.1  AGAATTCAAC TTCGC-GTTG GC--CATTTT TTGCTTAATG GTGTGGGACC TCCTTTCTCT TTCTCTCTCT CTCCGTGTAT ATGCAAGTGT
OY748513.1  AGAATTCAAC TTCGC-GTTG GC--CATTTT TTGCTTAATG GTGTGGGACC TCCTTTCTCT TTCTCTCTCT CTCCGTGTAT ATGCAAGTGT
OY748513.1  AGAATTCAAC TTCGC-GTTG GC--CATTTT TTGCTTAATG GTGTGGGACC TCCTTTCTCT TTCTCTCTCT CTCCGTGTAT ATGCAAGTGT
FJ753378.1  AGAATTCAAC TTCGC-GTTG GC--CATTTT TTGCTTAATG GTGTGGGACC TCCTTTCTCT TTCTCTCTCT CTCCGTGTAT ATGCAAGTGT
FJ753380.1  AGAATTCAAC TTCGC-GTTG GC--CATTTT TTGCTTAATG GTGTGGGACC TCCTTTCTCT TTCTCTCTCT CTCCGTGTAT ATGCAAGTGT
FJ753379.1  AGAATTCAAC TTCGC-GTTG GC--CATTTT TTGCTTAATG GTGTGGGACC TCCTTTCTCT TTCTATCTCT CTCCGTGTAT ATGCAAGTGT
LS997626.2  AGAATTCAAC TTCGC-GTTG GC--CATTTT TTGCTTAATG GTGTGGGACC TCCTTTCTCT TTCTCTCTCT CTCCGTGTAT ATGCAAGTGT
LS997626.2  AGAATTCAAC TTCGC-GTTG GC--CATTTT TTGCTTAATG GTGTGGGACC TCCTTTCTCT TTCTCTCTCT CTCCGTGTAT ATGCAAGTGT
LS997626.2  AGAATTCAAC TTCGC-GTTG GC--CATTTT TTGCTTAATG GTGTGGGACC TCCTTTCTCT TTCTCTCTCT CTCCGTGTAT ATGCAAGTGT
LS997626.2  AGAATTCAAC TTCGC-GTTG GC--CATTTT TTGCTTAATG GTGTGGGACC TCCTTTCTCT TTCTCTCTCT CTCCGTGTAT ATGCAAGTGT
LS997626.2  AGAATTCAAC TTCGC-GTTG GC--CATTTT TTGCTTAATG GTGTGGGACC TCCTTTCTCT TTCTCTCTCT CTCCGTGTAT ATGCAAGTGT
LS997626.2  AGAATTCAAC TTCGC-GTTG GC--CATTTT TTGCTTAATG GTGTGGGACC TCCTTTCTCT TTCTCTCTCT CTCCGTGTAT ATGCAAGTGT
MT940887.1  AGAATTCAAC TTCGC-GTTG GC--CATTTT TTGCTTAATG GTGTGGGACC TCCTTTCTCT TTCTCTCTCT CTCCGTGTAT ATGCAAGTGT
FJ753382.1  AGAATTCAAC TTCGC-GTTG GC--CATTTT TTGCTTAATG GTGTGGGACC TCCTTTCTCT TTCTCTCTCT CTCCGTGTAT ATGCAAGTGT
FJ753377.1  AGAATTCAAC TTCGC-GTTG GC--CATTTT TTGCTTAATG GTGTGGGACC TCCTTTCTCT TTCTCTCTCT CTCCGTGTAT ATGCAAGTGT
OY748421.1  AGAATTCAAC TTCGC-GTTG GC--CATTTT TTGCTTAATG GTGTGGGACC TCCTTTCTCT TTCTCTCTCT CTCCGTGTAT ATGCAAGTGT
OY748421.1  AGAATTCAAC TTCGC-GTTG GC--CATTTT TTGCTTAATG GTGTGGGACC TCCTTTCTCT TTCTCTCTCT CTCCGTGTAT ATGCAAGTGT
OY748421.1  AGAATTCAAC TTCGC-GTTG GC--CATTTT TTGCTTAATG GTGTGGGACC TCCTTTCTCT TTCTCTCTCT CTCCGTGTAT ATGCAAGTGT
FJ753376.1  AGAATTCAAC TTCGC-GTTG GC--CATTTT TTGCTTAATG GTGTGGGACC TCCTTTCTCT TTCTATCTCT CTCCGTGTAT ATGCAAGTGT
MW538634.1  AGAATTCAAC TTCGC-GTTG GC--CATTTT TTGCTTAATG GTGTGGGACC TCCTTTCTCT TTCTCTCTCT CTCCGTGTAT ATGCAAGTGT
MT940883.1  AGAATTCAAC TTCGC-GTTG GC--CATTTT TTGCTTAATG GTGTGGGACC TCCTTTCTCT TTCTCTCTCT CTCCGTGTAT ATGCAAGTGT
```

```
MT497968.1  AGAATTCAAC TTCGC-GTTG GC--CATTTT TTGCTTAATG GTGTGGGACC TCCTTTCTCT TTCTCTCTCT CTCCGTGTAT ATGCAAGTGT
JQ061322.1  AGAATTCAAC TTCGC-GTTG GC--CATTTT TTGCTTAATG GTGTGGGACC TCCTTTCTCT TTCTCTCTCT CTCCGTGTAT ATGCAAGTGT
MT940886.1  AGAATTCAAC TTCGC-GTTG GC--CATTTT TTGCTTAATG GTGTGGGACC TCCTTTCTCT TTCTCTCTCT CTCCGTGTAT ATGCAAGTGT
MT940877.1  AGAATTCAAC TTCGC-GTTG GC--CATTTT TTGCTTAATG GTGTGGGACC TCCTTTCTCT TTCTCTCTCT CTCCGTGTAT ATGCAAGTGT
MT940888.1  AGAATTCAAC TTCGC-GTTG GC--CATTTT TTGCTTAATG GTGTGGGACC TCCTTTCTCT TTCTCTCTCT CTCCGTGTAT ATGCAAGTGT
AJ300484.1  AGAATTCAAC TTCGC-GTTG GC--CATTTT TTGCTTAATG GTGTGGGACC TCCTTTCTCT TTCTCTCTCT CTCCGTGTAT ATGCAAGTGT
MT497927.1  AGAATTCAAC TTCGC-GTTG GC--CATTTT TTGCTTAATG GTGTGGGACC TCCTTTCTCT TTCT--CTCT CTCCGTGTAT ATGCAAGTGT
MT497929.1  AGAATTCAAC TTCGC-GTTG GC--CATTTT TTGCTTAATG GTGTGGGACC TCCTTTCTCT TTCTCTCTCT CTCCGTGTAT ATGCAAGTGT
FJ753383.1  AGAATTCAAC TTCGC-GTTG GC--CATTTT TTGCTTAATG GTGTGGGACC TCCTTTCTCT TTCTCTCTCT CTCCGTGTAT ATGCAAGTGT
MT497941.1  AGAATTCAAC TTCGC-GTTG GC--CATTTT TTGCTTAATG GTGTGGGACC TCCTTTCTCT TTCTCTCTCT CTCCGTGTAT ATGCAAGTGT
FJ753384.1  AGAATTCAAC TTCGC-GTTG GC--CATTTT TTGCTTAATG GTGTGGGACC TCCTTTCTCT TTCTCTCTCT CTCCGTGTAT ATGCAAGTGT
MT940880.1  AGAATTCAAC TTCGC-GTTG GC--CATTTT TTGCTTAATG GTGTGGGACC TCCTTTCTCT TTCTCTCTCT CTCCGTGTAT ATGCAAGTGT
FJ753374.1  AGAATTCAAC TTCGC-GTTG GC--CATTTT TTGCTTAATG GTGTGGGACC TCCTTTCTCT TTCTCTCTCT CTCCGTGTAT ATGCAAGTGT
FJ753375.1  AGAATTCAAC TTCGC-GTTG GC--CATTTT TTGCTTAATG GTGTGGGACC TCCTTTCTCT TTCTCTCTCT CTCCGTGTAT ATGCAAGTGT
FJ753381.1  AGAATTCAAC TTCGC-GTTG GC--CATTTT TTGCTTAATG GTGTGGGACC TCCTTTCTCT TTCTCTCTCT CTCCGTGTAT ATGCAAGTGT
MT497946.1  AGAATTCAAC TTCGC-GTTG GC--CATTTT TTGCTTAATG GTGTGGGACC TCCTTTCTCT TTCTCTCTCT CTCCGTGTAT ATGCAAGTGT
AJ300483.1  AGAATTCAAC TTCGCTGTTG GCTCCATTTT TTGCTTAATG GTGTGGGACC TCCTTTCTCT TTCTCTCTCT CTCCGTGTAT ATGCAAGTGT
FJ753385.1  AGAATTCAAC TTCGC-GTTG GC--CATTTT TTGCTTAATG GTGTGGGACC TCCTTTCTCT TTCTCTCTCT CTCCGTGTAT ATGCAAGTGT
Lbraz-F     ---------- ---------- ---------- ---------- ---------- ---------- ---------- ---------- ----------
Lbraz-R     ---------- ---------- ---------- ---------- ---------- ---------- ---------- ---------- --GCAAGTGT
            ....|....| ....|....| ....|....| ....|....| ....|....| ....|....| ....|....| ....|....| ....|....|
              455       465       475       485       495       505       515       525       535
MT497975.1  GTGTGGTTCT CTATACATA- ---GAGAACC GCTCACGCAA AAACATACTC AGAGAAGAGG GGGGAAGACG AGAGGGGGGG -AGCCCCACC
MT940879.1  GTGTGGTTCT CTATACATA- ---GAGAACC GCTCACGCAA AAACATACTC AGAGAAGAGG GGGGAAGACG AGAGGGGGGG -AGCCCCACC
MT940876.1  GTGTGGTTCT CTATACATA- ---GAGAACC GCTCACGCAA AAACATACTC AGAGAAGAGG GGGGAAGACG AGAGGGGGGG -AGCCCCACC
OY748513.1  GTGTGGTTCT CTATACATA- ---GAGAACC GCTCACGCAA AAACATACTC AGAGAAGAGG GGGGAAGACG AGAGGGGGGG -AGCCCCACC
OY748513.1  GTGTGGTTCT CTATACATA- ---GAGAACC GCTCACGCAA AAACATACTC AGAGAAGAGG GGGGAAGACG AGAGGGGGGG -AGCCCCACC
FJ753378.1  GTGTGGTTCT CTATACATA- ---GAGAACC GCTCACGCAA AAACATACTC AGAGAAGAGG GGGGAAGACG AGAGGGGGGG -AGCCCCACC
FJ753380.1  GTGTGGTTCT CTATACATA- ---GAGAACC GCTCACGCAA AAACATACTC AGAGAAGAGG GGGGAAGACG AGAGGGGGGG -AGCCCCACC
```

FJ753379.1 **GTGTGGTTCT CTATAC**ATA- ---GAGAACC GCTCACGCAA AAACATACTC AGAGAAGAGG GGGGAAGACG AGAGGGGGGG -AGCCCCACC
LS997626.2 **GTGTGGTTCT CTATAC**ATA- ---GAGAACC GCTCACGCAA AAACATACTC AGAGAAGAGG GGGGAAGACG AGAGGGGGGG -AGCCCCACC
LS997626.2 **GTGTGGTTCT CTATAC**ATA- ---GAGAACC GCTCACGCAA AAACATACTC AGAGAAGAGG GGG-AAGACG AGAGGGGGGG -AGCCCCACC
LS997626.2 **GTGTGGTTCT CTATAC**ATA- ---GAGAACC GCTCACGCAA AAACATACTC AGAGAAGAGG GGGGAAGACG AGAGGGGGG- -AGCCCCACC
LS997626.2 **GTGTGGTTCT CTATAC**ATA- ---GAGAACC GCTCACGCAA AAACATACTC AGAGAAGAGG GGGGAAGACG AGAGGGGGG- -AGCCCCACC
LS997626.2 **GTGTGGTTCT CTATAC**ATA- ---GAGAACC GCTCACGCAA AAACATACTC AGAGAAGAGG GGGGAAGACG AGAGGGGGGG GAGCCCCACC
LS997626.2 **GTGTGGTTCT CTATAC**ATA- ---GAGAACC GCTCACGCAA AAACATACTC AGAGAAGAGG GGG-AAGACG AGAGGGGGG- -AGCCCCACC
MT940887.1 **GTGTGGTTCT CTATAC**ATA- ---GAGAACC GCTCACGCAA AAACATACTC AGAGAAGAGG GGGGAAGACG AGAGGGGGGG -AGCCCCACC
FJ753382.1 **GTGTGGTTCT CTATAC**ATA- ---GAGAACC GCTCACGCAA AAACATACTC AGAGAAGAGG GGGGAAGACG AGAGGGGGGG -AGCCCCACC
FJ753377.1 **GTGTGGTTCT CTATAC**ATA- ---GAGAACC GCTCACGCAA AAACATACTC AGAGAAGAGG GGGGAAGACG AGAGGGGGGG -AGCCCCACC
OY748421.1 **GTGTGGTTCT CTATAC**ATA- ---GAGAACC GCTCACGCAA AAACATACTC AGAGAAGAGG GGGGAAGACG AGAGGGGGGG -AGCCCCACC
OY748421.1 **GTGTGGTTCT CTATAC**ATA- ---GAGAACC GCTCACGCAA AAACATACTC AGAGAAGAGG GGGGAAGACG AGAGGGGGGG -AGCCCCACC
OY748421.1 **GTGTGGTTCT CTATAC**ATA- ---GAGAACC GCTCACGCAA AAACATACTC AGAGAAGAGG GGGGAAGACG AGAGGGGGGG -AGCCCCACC
FJ753376.1 **GTGTGGTTCT CTATAC**ATA- ---GAGAACC GCTCACGCAA AAACATACTC AGAGAAGAGG GGGGAAGACG AGAGGGGGGG -AGCCCCACC
MW538634.1 **GTGTGGTTCT CTATAC**ATAC ATAGAGAACC GCTCACGCAA AAACATACTC AGAGAAGAGG GGGGAAGACG AGAGGGGGGG -AGCCCCACC
MT940883.1 **GTGTGGTTCT CTATAC**ATAC ATAGAGAACC GCTCACGCAA AAACATACTC AGAGAAGAGG GGGGAAGACG AGAGGGGGGG -AGCCCCACC
MT497968.1 **GTGTGGTTCT CTATAC**ATAC ATAGAGAACC GCTCACGCAA AAACATACTC AGAGAAGAGG GGG-AAGACG AGAGGGGGGG GAGCCCCACC
JQ061322.1 **GTGTGGTTCT CTATAC**ATAC ATAGAGAACC GCTCACGCAA AAACATACTC AGAGAAGAGG GGGGAAGACG AGAGGGGGGG -AGCCCCACC
MT940886.1 **GTGTGGTTCT CTATAC**ATAC ATAGAGAACC GCTCACGCAA AAACATACTC AGAGAAGAGG GGGGAAGACG AGAGGGGGGG -AGCCCCACC
MT940877.1 **GTGTGGTTCT CTATAC**ATAC ATAGAGAACC GCTCACGCAA AAACATACTC AGAGAAGAGG GGGGAAGACG AGAGGGGAGG -AGCCCCACC
MT940888.1 **GTGTGGTTCT CTATAC**ATA- ---GAGAACC GCTCACGCAA AAACATACTC AGAGAAGAGG GGGGAAGACG AGAGGGGGGG -AGCCCCACC
AJ300484.1 **GTGTGGTTCT CTATAC**ATAC ATAGAGAACC GCTCACGCAA AAACATACTC AGAGAAGAGG GGGGAAGACG AGAGGGGGGG AGCCCCACCA
MT497927.1 **GTGTGGTTCT CTATAC**ATA- ---GAGAACC GCTCACGCAA AAACATACTC AGAGAAGAGG GGGGAAGACG AGAGGGGGGG -AGCCCCACC
MT497929.1 **GTGTGGTTCT CTATAC**ATAC ATAGAGAACC GCTCACGCAA AAACATACTC AGAGAAGAGG GGG-AAGACG AGAGGGGGGG GAGCCCCACC
FJ753383.1 **GTGTGGTTCT CTATAC**ATAC ATAGAGAACC GCTCACGCAA AAACATACTC AGAGAAGAGG GGGGAAGACG AGAGGGGGGG -AGCCCCACC
MT497941.1 **GTGTGGTTCT CTATAC**ATA- ---GAGAACC GCTCACGCAA AAACATACTC AGAGAAGAGG GGG-AAGACG AGAGGGGGGG GAGCCCCACC
FJ753384.1 **GTGTGGTTCT CTATAC**ATAC ATAGAGAACC GCTCACGCAA AAACATACTC AGAGAAGAGG GGGGAAGACG AGAGGGGGGG -AGCCCCACC
MT940880.1 **GTGTGGTTCT CTATAT**ATAC ATAGAGAACC GCTCACGCAA AAACATACTC AGAGAAGAGG GGGGAAGACG AGAGGGGGGG GAGCCCCACC
FJ753374.1 **GTGTGGTTCT CTATAC**ATAC ATAGAGAACC GCTCACGCAA AAACATACTC AGAGAAGAGG GGGGAAGACG AGAGGGGGGG -AGCCCCACC

https://doi.org/10.3390/diagnostics16111704

FJ753375.1 **GTGTGGTTCT CTATAC**ATAC ATAGAGAACC GCTCACGCAA AAACATACTC AGAGAAGAGG GGGGAAGACG AGAGGGGGGG -AGCCCCACC
FJ753381.1 **GTGTGGTTCT CTATAC**ATAC ATAGAGAACC GCTCACGCAA AAACATACTC AGAGAAGAGG GGGGAAGACG AGAGGGGGGG -AGCCCCACC
MT497946.1 **GTGTGGTTCT CTATAT**ATAC ATAGAGAACC GCTCACGCAA AAACATACTC AGAGAAGAGG G--GAAGACG AGAGGGGGGG GAACCCCACC
AJ300483.1 **GTGTGGTTCT CTATAC**ATA- ---GAGAACC GCTCACGCAA AAACATACTC AGAGAAGAGG GGGGAAGACG AGAGGGGGGG -AGCCCCACC
FJ753385.1 **GTGTGGTTCT CTATAC**ATAC ATAGAGAACC GCTCACGCAA AAACATACTC AGAGAAGAGG GGGGAAGACG AGAGGGGGGG -AGCCCCACC
Lbraz-F ---------- ---------- ---------- ---------- ---------- ---------- ---------- ---------- ----------
Lbraz-R **GTGTGGTTCT CTATAC**---- ---------- ---------- ---------- ---------- ---------- ---------- ----------
....|....| ....
545
MT497975.1 ACAAACCTTT GTTT
MT940879.1 ACAAACCTTT GTTT
MT940876.1 ACAAACCTTT GTTT
OY748513.1 ACAAACCTTT GTTT
OY748513.1 ACAAACCTTT GTTT
FJ753378.1 ACAAACCTTT GTTT
FJ753380.1 ACAAACCTTT GTTT
FJ753379.1 ACAAACCTTT GTTT
LS997626.2 ACAAACCTTT GTTT
LS997626.2 ACAAACCTTT GTTT
LS997626.2 ACAAACCTTT GTTT
LS997626.2 ACAAACCTTT GTTT
LS997626.2 ACAAACCTTT GTTT
LS997626.2 ACAAACCTTT GTTT
MT940887.1 ACAAACCTTT GTTT
FJ753382.1 ACAAACCTTT GTTT
FJ753377.1 ACAAACCTTT GTTT
OY748421.1 ACAAACCTTT GTTT
OY748421.1 ACAAACCTTT GTTT
OY748421.1 ACAAACCTTT GTTT

https://doi.org/10.3390/diagnostics16111704

```
FJ753376.1   ACAAACCTTT GTTT
MW538634.1   ACAAACCTTT GTTT
MT940883.1   ACAAACCTTT GTTT
MT497968.1   ACAAACCTTT GTTT
JQ061322.1   ACAAACCTTT GTTT
MT940886.1   ACAAACCTTT GTTT
MT940877.1   ACAAACCTTT GTTT
MT940888.1   ACAAACCTTT GTTT
AJ300484.1   CAAACCTTTG TTT-
MT497927.1   ACAAACCTTT GTTT
MT497929.1   ACAAACCTTT GTTT
FJ753383.1   ACAAACCTTT GTTT
MT497941.1   ACAAACCTTT GTTT
FJ753384.1   ACAAACCTTT GTTT
MT940880.1   ACAAACCTTT GTTT
FJ753374.1   ACAAACCTTT GTTT
FJ753375.1   ACAAACCTTT GTTT
FJ753381.1   ACAAACCTTT GTTT
MT497946.1   ACAAACCTTT GTTT
AJ300483.1   ACAAACCTTT GTTT
FJ753385.1   ACAAACCTTT GTTT
Lbraz-F      ---------- ----
Lbraz-R      ---------- ----
```

diagnostics MDPI

Supplementary Table S2: Consensus nucleotide sequences of target regions used in melting curve-based real-time PCR (Leish-qPCR). To obtain positive controls, the consensus sequences of each target were inserted into the pUC57 plasmid (FastBio Ltda., Ribeirão Preto, Brazil).

***Leishmania* kDNA consensus nucleotide sequence**
GKAGGGGCGTTCTGCGRRWWYSGRWWWWTBGVWWRCAGAAAYCCCGTTCAWAAW-<br>WTSCVBSRAWWWTSBYRWWWWTBRVSYYSSVSSBBSVMAAYTRGGGGTTGGTGTAAWATAGG
***L.* (*L.*) *amazonensis* ITS2 consensus nucleotide sequence**
TGGGCTCTCTCTCTCTGTTATGTGTGTGGTATATACATATTATATATATTAGAG-<br>TAGGTGTGTGTGTGTGTATGTGTTTTACACATATATATTATATGCACCCTCACTCTCTCATGTATAATATATATACACACACACG<br>CAGAGAAAAAAGAGAGGGTTCCTGTGTGCTCTCCCCGCGCACCCCCGACAACCTTTGTTTACAGACCTGAGTGTTTGGC
***L.* (*V.*) *braziliensis* ITS2 consensus nucleotide sequence**
GCAGTCTCTCTCTCTCCTCTCTCCTCTCTATTCTCTCTTCTCCTCTCACTTTAAGAGGGGG-<br>GAGAGGAGGGGTTTTATATAGAGAAGAGGGGTGTGGGGGGGGGGAGGCTGTGACCAGGATAATTAAACAAAAAACCAAAACGAGA<br>ATTCAACTTCGCTGTTGGCTCCATTTTTTGCTTAATGGTGTGGGAC-<br>CTCCTTTCTCTTTCTCTCTCTCTCCGTGTATATGCAAGTGTGTGTGGTTCTCTATAC

Supplementary Table S3. Performance of Leish-qPCR in clinical samples.

Samples	Microscopic analysis[a]	Leish-qPCR
01	Negative	*L.* (*L.*) *amazonensis*
02	Negative	Negative
03	Negative	Negative
04	Negative	Negative
05	Positive	*Leishmania* spp.
06	Negative	Negative
07	Negative	*L.* (*V.*) *braziliensis*
08	Negative	Negative
09	Negative	Negative
10	Positive	*L.* (*V.*) *braziliensis*
11	Positive	*Leishmania* spp.
12	Negative	Negative
13	Negative	Negative
14	Positive	*L.* (*L.*) *amazonensis*
15	Negative	Negative
16	Negative	*L.* (*L.*) *amazonensis*
17	Negative	Negative
18	Negative	Negative
19	Negative	Negative
20	Negative	Negative
21	Negative	Negative
22	Negative	Negative
23	Negative	Negative
24	Negative	Negative
25	Negative	Negative
26	Negative	Negative
27	Negative	Negative
28	Positive	*L.* (*V.*) *braziliensis*
29	Negative	Negative
30	Positive	*L.* (*V.*) *braziliensis*
31	Negative	Negative
32	Negative	Negative
33	Negative	Negative
34	Negative	Negative
35	Negative	Negative
36	Positive	*L.* (*V.*) *braziliensis*
37	Negative	*L.* (*V.*) *braziliensis*
38	Positive	*L.* (*V.*) *braziliensis*
39	Negative	*L.* (*V.*) *braziliensis*
40	Negative	*L.* (*V.*) *braziliensis*
41	Positive	*L.* (*V.*) *braziliensis*
42	Negative	*L.* (*V.*) *braziliensis*
43	Negative	*L.* (*V.*) *braziliensis*
44	Negative	Negative
45	Negative	*L.* (*V.*) *braziliensis*
46	Negative	*L.* (*V.*) *braziliensis*
47	Negative	Negative
48	Positive	*L.* (*V.*) *braziliensis*
49	Negative	*Leishmania* spp.
50	Negative	*L.* (*V.*) *braziliensis*

51	Negative	*L.* (*V.*) *braziliensis*
52	Positive	*L.* (*V.*) *braziliensis*
53	Negative	*L.* (*L.*) *amazonensis*
54	Positive	*L.* (*V.*) *braziliensis*
55	Positive	*L.* (*V.*) *braziliensis*
56	Negative	Negative
57	Negative	*L.* (*V.*) *braziliensis*
58	Negative	Negative
59	Positive	*L.* (*V.*) *braziliensis*
60	Negative	Negative

[a]Reference routine histopathological analysis of skin biopsy specimens collected from patients presenting with granulomatous lesions.

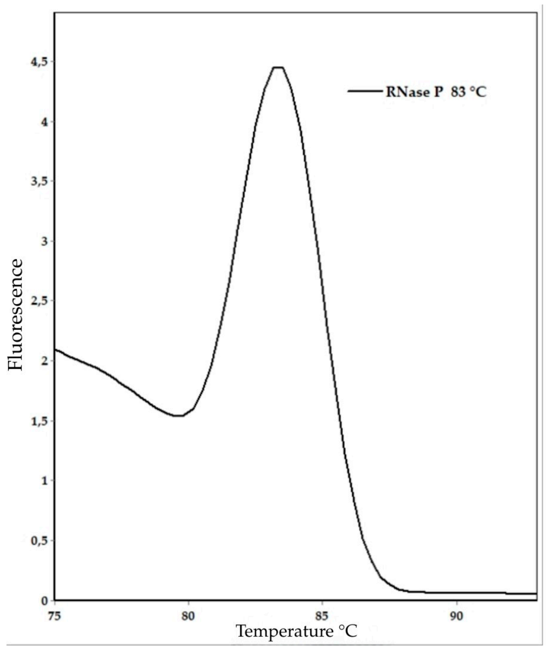

Supplementary Figure S1: Melting Curves analysis showing the melting peak (Tm) of RNAse P amplicon using monoplex qPCR assays.

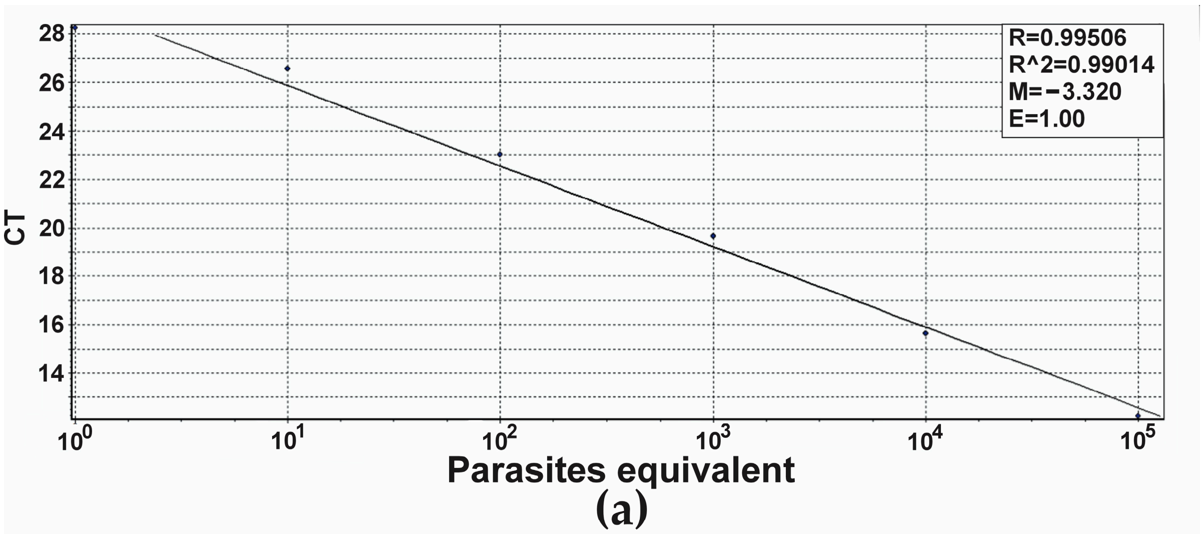

(a)

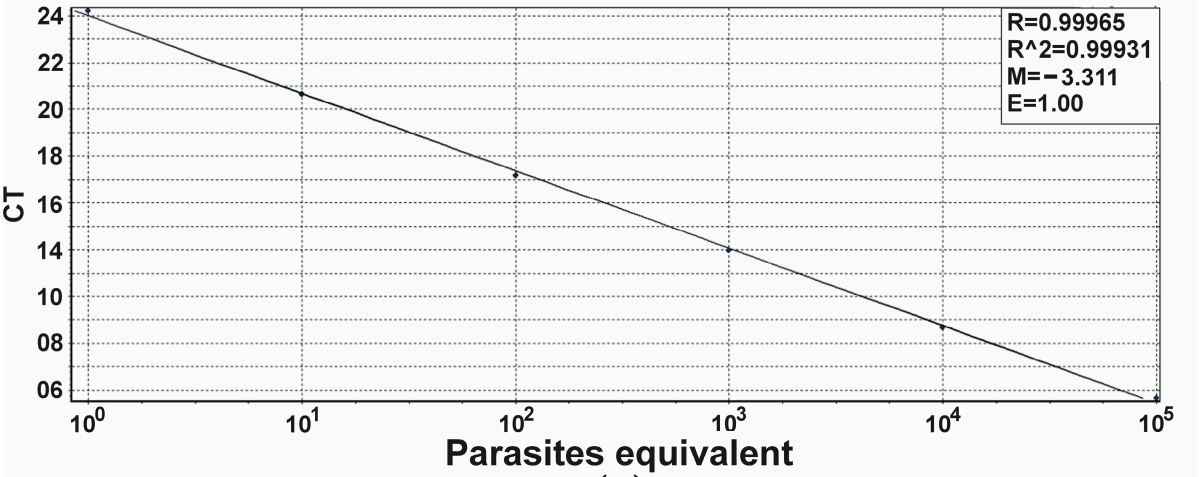

(a)

Supplementary Figure S2. Sensitivity of Leish-qPCR assays. kDNA minicircle of (**a**) *L.* (*L.*) *amazonensis* and (**b**) *L.* (*V.*) *braziliensis*. Amplification plots of 10-fold serial dilutions corresponding to 10^0–10^5 parasite equivalent numbers. Standard curves were generated by linear regression analysis of threshold cycle (Ct) versus parasite equivalent numbers. The slope (M), regression coefficient (R), and amplification efficiency of the qPCR assay are shown (**a**,**b**). The analyses were performed using the Rotor-Gene Q Series instrument and processed with the Rotor-Gene Q Series Software version 2.1.0.9. based on the Ct values obtained during amplification.

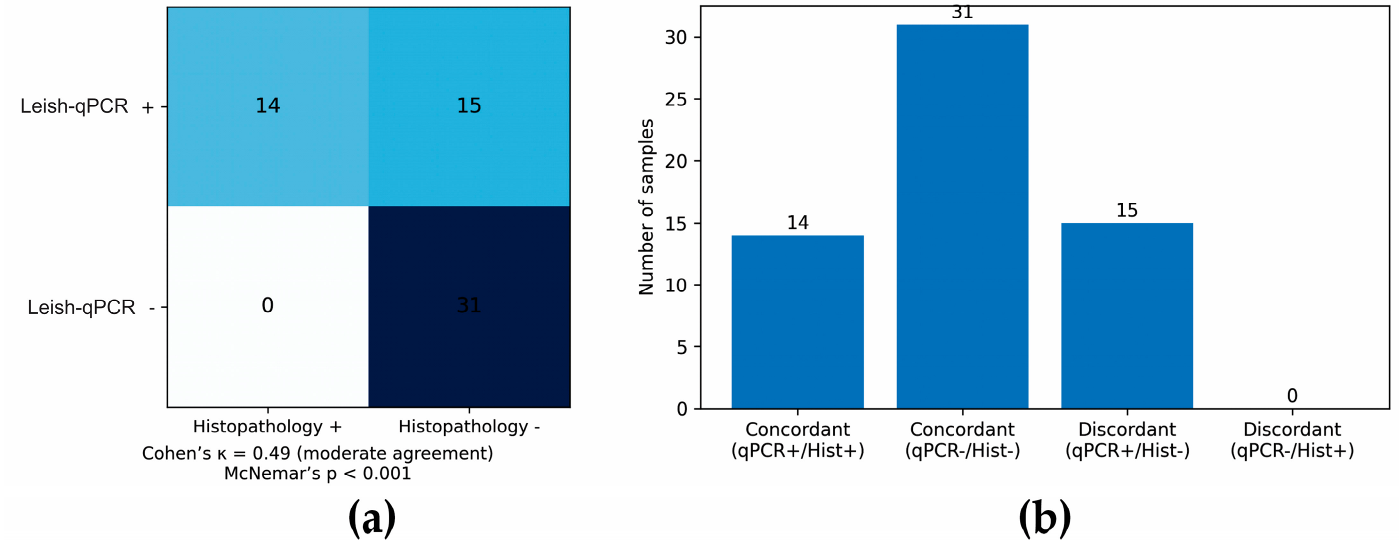

Supplementary Figure S3. Statistical agreement between histopathological diagnosis and the Leish-qPCR, using Cohen's Kappa coefficient. (**a**) Confusion matrix of Leish-qPCR versus histopathological examination. (**b**) Concordance/Discordance distribution. The analysis was implemented in Python version 3.13 using the sklearn.metrics library, ensuring a robust quantitative assessment of diagnostic reliability.
